# Longitudinal changes in blood biomarkers and their ability to predict type 2 diabetes mellitus—The Tromsø study

**DOI:** 10.1002/edm2.325

**Published:** 2022-02-11

**Authors:** Giovanni Allaoui, Charlotta Rylander, Maria Averina, Tom Wilsgaard, Ole‐Martin Fuskevåg, Vivian Berg

**Affiliations:** ^1^ Division of Diagnostic Services Department of Laboratory Medicine University Hospital of North Norway Tromsø Norway; ^2^ Department of Medical Biology Faculty of Health Sciences UiT‐The Arctic University of Norway Tromsø Norway; ^3^ Department of Community Medicine Faculty of Health Sciences UIT‐The Arctic University of Norway Tromsø Norway

**Keywords:** biomarkers, blood test, health service, longitudinal survey, preventive, risk factors, type 2 diabetes mellitus

## Abstract

**Introduction:**

Identification of individuals at high risk of developing type 2 diabetes mellitus (T2DM) is important for early prevention of the disease. Once T2DM is established, it is difficult to treat and is associated with cardiovascular complications and increased mortality. We aimed to describe pre‐ and post‐diagnostic changes in blood biomarker concentrations over 30 years in individuals with and without T2DM, and to determine the predictive potential of pre‐diagnostic blood biomarkers.

**Methods:**

This nested case–control study included 234 participants in the Tromsø Study who gave blood samples at five time points between 1986 and 2016: 130 did not develop T2DM and were used as controls; 104 developed T2DM after the third time point and were included as cases. After stratifying by sex, we investigated changes in pre‐ and post‐diagnostic concentrations of lipids, thyroid hormones, HbA_1c_, glucose and gamma‐glutamyltransferase (GGT) using linear mixed models. We used logistic regression models and area under the receiver operating characteristic curve (AROC) to assess associations between blood biomarker concentrations and T2DM, as well as the predictive ability of blood biomarkers.

**Results:**

Cases and controls experienced different longitudinal changes in lipids, free T_3_, HbA_1c_, glucose, and GGT. The combination of selected blood biomarker concentrations and basic clinical information displayed excellent (AROC 0.78–0.95) predictive ability at all pre‐diagnostic time points. A prediction model that included HDL (for women), HbA_1c_, GGT, and basic clinical information demonstrated the strongest discrimination 7 years before diagnosis (AROC 0.95 for women, 0.85 for men).

**Conclusion:**

There were clear differences in blood biomarker concentrations between cases and controls throughout the study, and several blood biomarkers were associated with T2DM. Selected blood biomarkers (lipids, HbA_1c_, GGT) in combination with BMI, physical activity, elevated blood pressure, and family history of T2DM had excellent predictive ability 1–7 years before T2DM diagnosis and acceptable predictive ability up to 15 years before diagnosis.

## Graphical abstract

This nested case–control study with repeated measurements demonstrate clear differences in blood biomarker concentrations between cases and controls, and combining basic clinical information with biomarkers provides excellent type 2 diabetes prediction.

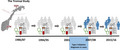

## 1 INTRODUCTION

The prevalence of type 2 diabetes mellitus (T2DM) has increased substantially over the past few decades and is one of the most important global health challenges of the 20th century.^1^ The disease is characterized by insufficient insulin secretion and/or insulin resistance and established risk factors include among other obesity, sedentary lifestyle, excess dietary intake, and genetic factors.^2^ Previous longitudinal studies of repeated pre‐diagnostic measurements have demonstrated increases in lipid and glucose concentrations 1.5–20 years before T2DM diagnosis, with steeper increases closer to diagnosis.^3^, ^4^, ^5^, ^6^, ^7^, ^8^, ^9^, ^10^ Thus, disruption of metabolic homeostasis involving lipids, thyroid hormones, glucose, and liver enzymes is associated with T2DM.^5^, ^8^, ^9^, ^11^, ^12^, ^13^ However, the sequence of this disruption and its relative contribution to the progression from normal to impaired glucose tolerance, and ultimately to T2DM, remains unknown.^14^, ^15^

Prediabetes (i.e., higher‐than‐normal blood glucose concentrations) precedes T2DM. Once T2DM has manifested, it is irreversible, difficult to treat, and associated with cardiovascular complications and increased mortality.^16^, ^17^, ^18^ The identification of blood biomarkers and the development of risk score models for prediabetes and T2DM are therefore highly relevant, as they will enable early identification of high‐risk individuals. There are currently many risk score models for diabetes (reviewed by Buijsse et al.^19^) most are based on basic clinical information like age, body mass index (BMI), physical activity, blood pressure and genetic predisposition, but some also include blood biomarkers. For instance, the FINDRISC (including basic clinical information as well as daily consumption of vegetables, fruits or berries, and history of high glucose) and the Framingham (including basic clinical information as well as high‐density lipoprotein (HDL) and triglycerides) risk scores for diabetes have been shown to successfully identify high‐risk individuals 5–7 years before diagnosis.^20^, ^21^

Several studies of risk score models have shown that adding blood biomarkers to basic clinical information improves predictive ability,^4^, ^20^, ^22^ especially biomarkers involved in glycaemic processes, uric acid, and lipids. However, most studies on prediction models are based on a single baseline blood sample.^4^, ^23^ The Tromsø Study contains blood biomarker concentrations and basic clinical information for up to five time points. Hence, we aimed to describe pre‐ and post‐diagnostic changes in blood biomarker concentrations over 30 years in individuals with and without T2DM, and to determine the predictive potential of pre‐diagnostic blood biomarkers.

## 2 METHODS

### 2.1 Study population

The Tromsø Study is a population‐based health survey carried out in the Tromsø municipality in Northern Norway. The first survey, Tromsø1, was carried out in 1974, and six more surveys followed (Tromsø2‐Tromsø7), one about every 6–7 years. During each survey, participants completed questionnaires, underwent a clinical examination and gave a blood sample.^24^, ^25^

The present, longitudinal, nested case–control study includes blood samples collected from the same individuals at five time points: Tromsø3 (1986/87), Tromsø4 (1994/95), Tromsø5 (2001), Tromsø6 (2007/08) and Tromsø7 (2015/16). Hereafter, Tromsø3‐Tromsø7 will be referred to as time point 1–5 (T1–T5), where cases developed T2DM after T3. Hence, T1–T3 was defined as the pre‐diagnostic time period, whereas T4 and T5 were defined as the post‐diagnostic time period.

**FIGURE 1:**
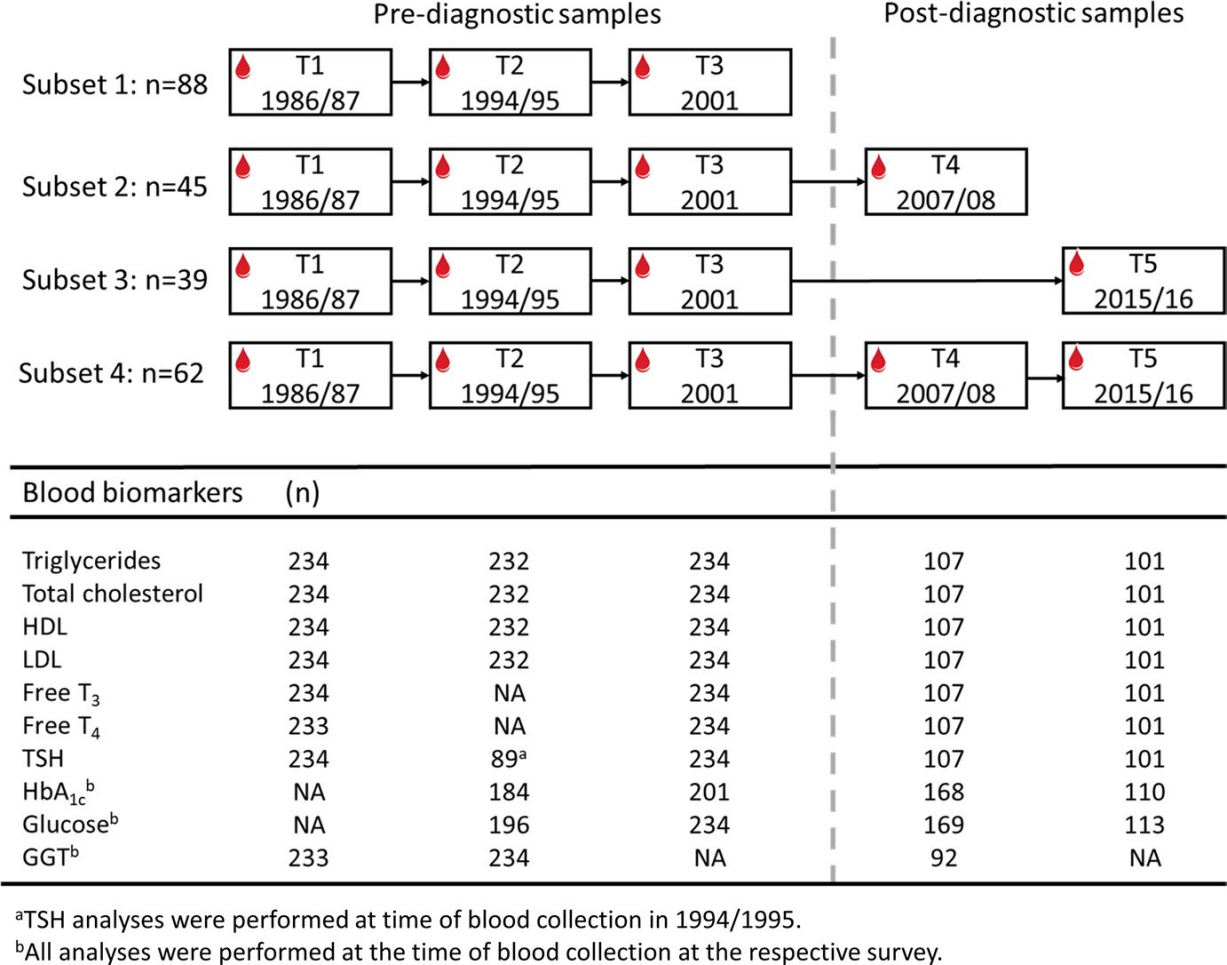
Study flow chart presents the study sample according to participation in three or more surveys, and how many blood samples were analysed for the different biomarkers at each time point (T1–T5). HbA_1c_, Glycated haemoglobin; HDL, High‐density lipoprotein; GGT, Gamma‐glutamyltransferase; LDL, Low‐density lipoprotein; NA, not available; T, Time point; T_3_, Triiodothyronine; T_4,_ Thyroxine; T2DM, type 2 diabetes mellitus; TSH, Thyroid‐stimulating hormone. The Tromsø Study 1986–2016

Initially, all participants with a T2DM diagnosis were recorded in a local diabetes registry between 2000 (T3) and 2006 (T4), and available pre‐diagnostic serum samples were eligible for inclusion as cases (76 women, 69 men). We then randomly selected 76 women and 69 men who participated in the same surveys, had serum samples for T1–T3 and had no T2DM diagnosis recorded in a local diabetes registry during the surveys as controls. Of the initial 290 participants, we excluded 29 cases with glycated haemoglobin (HbA_1c_) ≥48 mmol/mol (6.5%) before or at T3, and seven controls with HbA_1c_ ≥48 mmol/mol (6.5%) at any time point. We also excluded participants who reported using medications that could affect glucose and thyroid hormone concentrations before T3 (8 controls, 2 cases). Thus, the final study population comprised 234 individuals (104 cases, 130 controls). Of these, 88 had blood samples for T1–T3 (38 cases, 50 controls), 45 (21 cases, 24 controls) had samples for T1–T4, 39 (18 cases, 21 controls) for T1–T3 and T5, and 62 (27 cases, 35 controls) had blood samples for T1–T5 (Figure 1). All participants gave informed consent at the time of each survey. The study protocol was approved by the Regional Ethics Committee, REK, nord (REK reference: 2015/1780/REK nord).

### 2.2 Questionnaires, clinical examination and blood collection

The Tromsø Study questionnaire and measurements have been described in detail elsewhere.^24^, ^25^ Briefly, each survey included a questionnaire that collected information on lifestyle habits, self‐reported diseases such as diabetes, family history of diseases including T2DM, parity and breastfeeding. A clinical examination was also conducted at each survey and included measurements of weight, height, waist circumference and blood pressure, among others, and the collection of non‐fasting blood samples. Several analyses were performed in fresh blood samples; serum samples were frozen and stored for later use.^25^

### 2.3 Laboratory analyses and availability of blood biomarkers

Serum samples were thawed and analysed for triglycerides, total cholesterol, low‐density lipoprotein (LDL), HDL, free triiodothyronine (T_3_), free thyroxine (T_4_) and thyroid‐stimulating hormone (TSH), but serum samples from T2 were insufficient for analyses of free T_3_, free T_4_ and TSH. Data from previous analyses carried out at the time of blood collection were available for TSH (T2), HbA_1c_ (T2–T5), glucose (T2–T5) and gamma‐glutamyltransferase (GGT; T1‐T2, T4). Included blood biomarkers varied at each time point (Figure 1). All analyses were performed at the University Hospital of North Norway, Department of Laboratory Medicine, using routine, established procedures. Serum concentrations of triglycerides, total cholesterol, LDL, HDL, free T_3_, free T_4_, TSH, glucose and GGT were determined using the Cobas^®^ 8000 platform (Roche Diagnostics, Switzerland). Until 2006, GGT was analysed at 37°C in a Hitachi 737 Automatic Analyser using commercial kits (Boehringer Manheim, Germany) according to the recommendations of the Scandinavian Enzymes Committee.^26^ HbA_1c_ was determined by high‐performance liquid chromatography using an automated analyser (Variant II, Bio‐Rad Laboratories). Laboratory personnel were blinded to the sample order and survey number. The laboratory is certified according to the ISO 151189 standard.^27^ Quality controls are run routinely, at three different concentrations every day, and the laboratory also participates in the external quality assessment program, Lab Quality.^28^ Total lipids (g/L) were calculated according to the formula^29^:

Totallipids=2.27×totalcholesterol+triglycerides+0.623

### 2.4 Statistical analyses

Blood biomarker concentrations and demographic variables are reported as means with standard deviations, medians with 5 and 95 percentiles, and/or frequencies with percentages. Sample characteristics were compared between cases and controls at each time point using unpaired two‐sample t‐tests for continuous variables and Pearson's chi‐squared for categorical variables.

Linear mixed effects models were used to explore the rate and significance of changes in blood biomarker concentrations at T1–T5, between and within cases and controls, after adjusting for the following established risk factors for T2DM^30^: age (continuous), BMI (continuous), physical activity (active: ≥3 h/week of light activity and/or ≥1 h hard exercise/week or sedentary: <3 h/week of activity that provoked transpiration or no activity), elevated blood pressure (systolic blood pressure ≥130, diastolic blood pressure ≥85, and/or if the subject was taking blood pressure medication, yes/no) and family history of T2DM (siblings and/or parents with T2DM, yes/no). Blood biomarkers were used as dependent variables (continuous), whereas T2DM status, established risk factors and indicator variables of time with interaction terms with T2DM status were used as independent variables. A random intercept at the participant level was included to control for repeated measurements over time, with an unstructured variance and covariance correlation structure for within‐group errors.

We assessed the associations between pre‐diagnostic blood biomarker concentrations and T2DM. Logistic regression analyses were used to estimate odds ratios of T2DM for each time point separately. We fitted two models per blood biomarker: the first included blood biomarker concentration as a continuous, independent variable; in the second model, the blood biomarker was dichotomized according to clinical guidelines and concentrations associated with an increased risk of T2DM. Both models were adjusted for established risk factors, and odds ratios were estimated either per 1‐unit increase in blood biomarker concentration or above versus below the defined clinical cut‐off values: triglycerides >1.70 g/L, HDL <1.30 mmol/L for women and <1.03 for men,^30^ total cholesterol >5.00 mmol/L, LDL >3.00 mmol/L^31^ and HbA_1c_ >39.0 mmol/mol (5.7%).^18^ Cut‐offs for blood biomarkers with no clinical guidelines were based on a receiver operating characteristics curve (ROC) analysis in pre‐diagnostic samples, which yielded the highest discrimination between cases and controls, and were as follows: total lipids >7.40 g/L for women (62.7% sensitivity, 63.3% specificity) and >7.59 for men (61.5% sensitivity, 61.5% specificity), free T_3_ >5.20 pmol/L for women (33.0% sensitivity, 80.4% specificity) and >5.12 for men (54.6% sensitivity, 59.0% specificity), free T_4_ <14.8 pmol/l for women (26.0% sensitivity, 53.6% specificity) and <14.0 for men (50.9% sensitivity, 34.7% specificity), TSH >1.92 mIU/L for women (47.0% sensitivity, 60.9 specificity) and >1.85 for men (61.1% sensitivity, 44.3% specificity), glucose >5.78 mmol/L for women (38.5% sensitivity, 91.3% specificity) and >5.59 for men (41.2% sensitivity, 77.3% specificity), and GGT >20.0 U/L for women (46.0% sensitivity, 83.9% specificity) and >25.0 for men (63.0% sensitivity, 68.9% specificity).

We assessed the following models: (1) a logistic regression model for established risk factors (age, BMI, physical activity, elevated blood pressure, family history of T2DM); (2) a blood biomarker model based on the significant blood biomarkers (*p *< .05) from the univariable unadjusted models, which were further reduced by a backwards selection process with best model fit as the selection criteria; and (3) a combined model including both established risk factors and blood biomarkers, using the same selection process as for the blood biomarker model. Model fit was assessed by Akaike's information criterion (AIC). Model discrimination was used to determine predictive value, assessed by area under the receiver operating characteristics (AROC). As per Hosmer and Lemeshow, an AROC of 0.50 indicates no discrimination, 0.50–0.70 poor discrimination, 0.70–0.80 acceptable discrimination, 0.80–0.90 excellent discrimination and ≥0.90 outstanding discrimination.^32^

Statistical analyses were performed in STATA (v. 17, StataCorp LLC, 4905 Lakeway Drive, College Station). All statistical analyses were stratified by sex, *p* values were two‐sided, and a 5% level of significance was used.

## 3 RESULTS

### 3.1 Study sample characteristics

Type 2 diabetes mellitus cases and controls were similar in age, whereas cases were heavier, had higher BMI, and larger waist circumference (except men at T5) at all time points (Table 1). At pre‐diagnostic time points, female cases had significantly higher blood pressure than controls, except for systolic blood pressure at T2. We observed no significant differences in blood pressure for males, except at T5, when cases had significantly lower diastolic blood pressure. In general, there were no differences in alcohol consumption or physical activity between cases and controls (Table S1), and no significant differences in parity or duration of breastfeeding between female cases and controls (Table 1). Female cases reported a family history of T2DM more frequently than female controls (Table S1).

**TABLE 1:** Characteristics of the study sample across five surveys of the Tromsø Study 1986–2016

			Pre‐diagnostic time points						Post‐diagnostic time points			
			T1 (1986/87)		T2 (1994/95)		T3 (2001)		T4 (2007/08)		T5 (2015/16)	
			Mean (SD)	ΔMean case‐control (95% CI)	Mean (SD)	ΔMean case‐control (95% CI)	Mean (SD)	ΔMean case‐control (95% CI)	Mean (SD)	ΔMean case‐control (95% CI)	Mean (SD)	ΔMean case‐control (95% CI)
Age (years)	Women^a^	Case	45.3 (6.31)	1.46 (−1.48, 4.39)	53.3 (6.31)	1.46 (−1.48, 4.39)	60.3 (6.31)	1.46 (−1.48, 4.39)	65.9 (6.39)	0.26 (−2.66, 3.18)	73.4 (6.07)	2.92 (−1.34, 7.19)
		Control	43.9 (8.98)		51.9 (8.98)		58.9 (8.98)		65.6 (7.83)		70.5 (9.71)	
	Men^b^	Case	48.4 (8.61)	2.05 (−1.57, 5.66)	56.4 (8.61)	2.05 (−1.57, 5.66)	63.4 (8.61)	2.05 (−1.57, 5.66)	68.5 (6.97)	1.61 (−2.37, 5.58)	72.6 (7.82)	2.34 (−3.43, 8.10)
		Control	46.4 (10.7)		54.4 (10.7)		61.4 (10.7)		66.9 (10.2)		70.2 (11.0)	
Parity (*n*)	Women^a^	Case	2.66 (1.56)	0.24 (−0.35, 0.83)	2.85 (1.38)	0.34 (−0.21, 0.89)	2.81 (1.54)	0.23 (−0.32, 0.79)	2.72 (1.45)	−0.09 (−0.70, 0.52)	2.62 (1.36)	−0.11 (−0.94, 0.73)
		Control	2.42 (1.56)		2.51 (1.45)		2.58 (1.45)		2.81 (1.52)		2.73 (1.83)	
Breastfeeding (months)	Women^a^	Case	NA	NA	13.5 (11.5)	−1.52 (−6.22, 3.19)	15.1 (12.2)	−0.71 (−5.59, 4.18)	14.7 (13.5)	0.22 (−8.41, 3.73)	11.4 (8.57)	−8.70 (−15.3, −2.10)*
		Control	NA	NA	15.0 (10.8)		15.8 (11.5)		17.0 (12.4)		20.1 (13.5)	
Weight (kg)	Women^a^	Case	71.9 (12.1)	8.41 (4.39, 12.4)***	77.5 (13.8)	10.7 (6.06, 15.4)***	81.9 (15.0)	12.2 (7.23, 17.2)***	81.7 (15.8)	12.6 (6.70, 18.5)***	81.6 (18.3)	11.8 (3.42, 20.2)**
		Control	63.4 (10.0)		66.7 (11.8)		69.7 (12.3)		69.1 (13.4)		69.8 (15.1)	
	Men^b^	Case	85.1 (12.9)	6.94 (2.81, 11.1)**	88.5 (13.3)	7.41 (3.07, 11.8)**	91.4 (14.0)	7.72 (2.98, 12.5)**	90.4 (12.0)	5.69 (0.41, 11.0)*	90.2 (14.5)	4.18 (−3.42, 11.8)
		Control	78.2 (9.24)		81.1 (10.2)		83.7 (11.6)		84.7 (10.9)		86.0 (11.7)	
BMI (kg/m^2^)	Women^a^	Case	27.5 (4.38)	3.63 (2.14, 5.13)***	29.8 (5.13)	4.54 (2.74, 6.34)***	31.8 (5.90)	5.22 (3.29, 7.15)***	31.8 (6.34)	5.05 (2.72, 7.37)***	31.5 (7.28)	4.64 (1.39, 7.89)**
		Control	23.9 (3.83)		25.3 (4.72)		26.5 (4.72)		26.7 (5.19)		26.9 (5.87)	
	Men^b^	Case	27.5 (3.55)	2.94 (1.77, 4.10)***	28.6 (3.49)	3.03 (1.83, 4.24)***	29.8 (3.58)	3.29 (1.99, 4.59)***	29.4 (3.45)	2.50 (0.95, 4.05)**	29.6 (3.79)	2.32 (0.19, 4.45)*
		Control	24.6 (2.73)		25.6 (3.03)		26.6 (3.46)		26.9 (3.29)		27.2 (3.49)	
Waist circumference (cm)	Women^a^	Case	NA	NA	93.0 (11.4)	11.8 (6.39, 17.3)***	96.1 (12.4)	12.0 (7.30, 16.8)***	103 (12.9)	12.7 (7.40, 17.9)***	105 (14.8)	14.7 (7.55, 21.8)***
		Control	NA	NA	81.2 (9.95)		84.1 (13.2)		90.1 (12.5)		90.0 (13.6)	
	Men^b^	Case	NA	NA	101 (7.89)	7.28 (4.19, 10.4)***	104 (9.43)	7.80 (4.23, 11.4)***	106 (8.66)	5.02 (0.81, 9.23)*	108 (12.5)	5.85 (−0.73, 12.4)
		Control	NA	NA	93.7 (7.37)		95.8 (9.87)		101 (9.36)		102 (10.1)	
Diastolic blood pressure (mmHg)	Women^a^	Case	81.3 (10.3)	6.14 (2.31, 9.97)**	83.9 (12.2)	4.93 (0.53, 9.33)*	85.1 (14.7)	6.97 (2.16, 11.8)**	79.2 (10.2)	2.37 (−1.86, 6.61)	71.0 (10.4)	−3.91 (−9.61, 1.80)
		Control	75.1 (10.5)		79.0 (11.8)	1.90 (−2.21, 6.01)	78.2 (11.7)		76.8 (10.5)		74.9 (11.9)	
	Men^b^	Case	85.6 (9.67)	3.36 (−0.32, 7.04)	85.7 (11.2)		83.2 (11.6)	−0.74 (−5.57, 4.09)	78.2 (12.0)	−4.22 (−9.33, 0.89)	72.8 (9.81)	−7.16 (−13.3, −1.05)*
		Control	82.2 (10.2)		83.8 (11.1)		84.0 (14.2)		82.4 (10.1)		80.0 (10.8)	
Systolic blood pressure (mmHg)	Women^a^	Case	131 (16.0)	7.49 (1.54, 13.4)*	142 (20.0)	7.36 (−0.09, 14.8)	146 (21.0)	11.3 (3.34, 19.2)**	154 (25.4)	6.39 (−4.22, 16.9)	140 (25.4)	0.96 (−11.5, 13.4)
		Control	124 (16.3)		135 (20.5)		135 (22.1)	1.26 (−6.40, 8.93)	147 (26.5)		139 (24.4)	
	Men^b^	Case	139 (14.2)	3.84 (−1.75, 9.43)	146 (19.8)	6.18 (−0.69, 13.0)	143 (20.5)		144 (24.4)	2.19 (−8.44, 12.8)	132 (18.1)	−7.58 (−18.7, 3.52)
		Control	135 (15.9)		139 (17.4)		142 (20.6)		142 (21.6)		139 (19.4)	

Abbreviations: BMI, body mass index; T, time point.

^a^ Fifty cases and 69 controls at T1–T3, 44 cases and 53 controls at T4, 26 cases and 40 controls at T5.

^b^ Fifty‐four cases and 61 controls at T1–T3, 38 cases and 38 controls at T4, 20 cases and 28 controls at T5.

* *p *< .05.

** *p *< .01.

*** *p *< .001.

**FIGURE 2:**
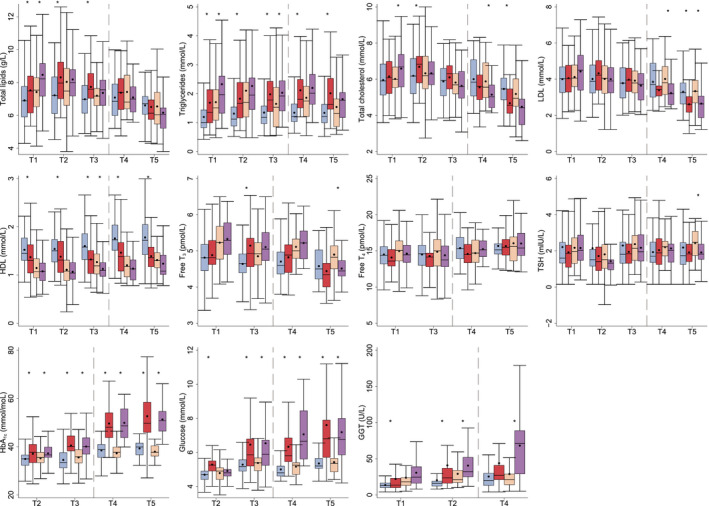
Pre‐ and post‐diagnostic blood biomarker concentrations across surveys in female cases (red) and controls (blue) and male cases (purple) and controls (orange). Sample number for females were: 50 cases and 69 controls at T1–T3, 44 cases and 53 controls at T4, 26 cases and 40 controls at T5; and for males: 54 cases and 61 controls at T1–T3, 38 cases and 38 controls at T4, 20 cases and 28 controls at T5. HbA_1c_, Glycated haemoglobin; HDL, High‐density lipoprotein; GGT, Gamma‐glutamyltransferase; LDL, Low‐density lipoprotein; T, Time point; T_3_, Triiodothyronine; T_4,_ Thyroxine; T2DM, type 2 diabetes mellitus; TSH, Thyroid‐stimulating hormone. The Tromsø Study 1986–2016

Female cases had significantly higher triglyceride, HbA_1c_, and glucose concentrations, and lower HDL concentrations than controls at all time points. Female cases also had significantly higher pre‐diagnostic total lipids, total cholesterol (T2), free T_3_ (T3) and GGT (T1–T2) concentrations than controls (Figure 2 and Table S2). However, post‐diagnostic total cholesterol and LDL concentrations were significantly lower in cases than controls. Similarly, male cases had higher HbA_1c_ and glucose (except at T2) concentrations than controls at all time points. Further, male cases had higher pre‐diagnostic total lipid (T1), triglyceride (T1 and T3), total cholesterol (T1), and GGT (T2) concentrations, and lower HDL concentrations (T3) than controls. Finally, post‐diagnostic total cholesterol (T4), free T_3_ (T5) and TSH (T5) concentrations were significantly higher in cases than controls.

### 3.2 Longitudinal changes in blood biomarkers

**FIGURE 3:**
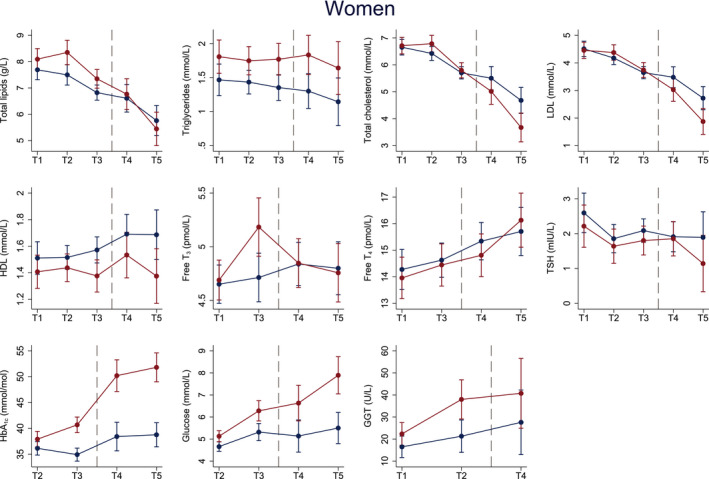
Estimated mean pre‐ and post‐diagnostic blood biomarker concentrations (y‐axis) across up to five time points (x‐axis) for female cases (red) and controls (blue). Sample numbers: 50 cases and 69 controls at T1–T3, 44 cases and 53 controls at T4, 26 cases and 40 controls at T5. Models are adjusted for age, BMI, physical activity, elevated blood pressure and family history of type 2 diabetes. Dots represent mean concentrations and whiskers the 95% CI around the mean. HbA_1c_, Glycated haemoglobin; HDL, High‐density lipoprotein; GGT, Gamma‐glutamyltransferase; LDL, Low‐density lipoprotein; NA, not available; T, Time point; T_3_, Triiodothyronine; T_4,_ Thyroxine; T2DM, type 2 diabetes mellitus; TSH, Thyroid‐stimulating hormone. The Tromsø Study 1986–2016

After adjusting for age, BMI, physical activity, elevated blood pressure and family history of T2DM, female cases experienced a significantly larger increase in pre‐diagnostic free T_3_ (T1–T3), HbA_1c_ (T2‐T3) and GGT (T1‐T2) concentrations compared to controls (Figure 3 and Table S3). Further, there was a significantly larger increase in HbA_1c_ concentrations, and a larger decrease in total cholesterol, LDL and free T_3_ concentrations in cases compared to controls from T3–T5.

**FIGURE 4:**
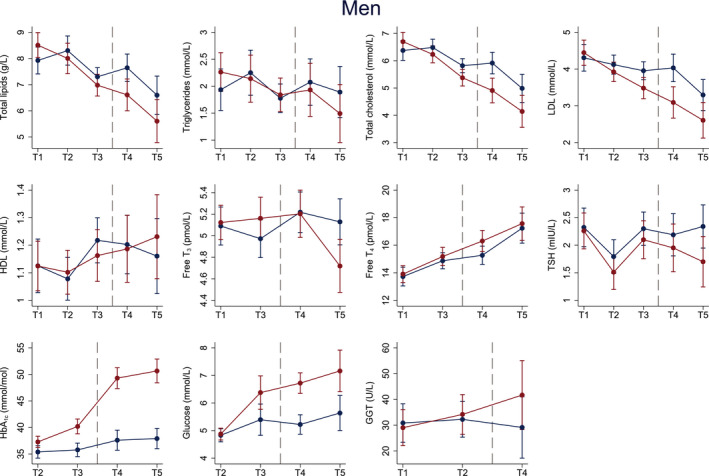
Estimated mean pre‐ and post‐diagnostic blood biomarker concentrations (y‐axis) across up to five time points (x‐axis) for male cases (red) and controls (blue). Sample numbers: 54 cases and 61 controls at T1–T3, 38 cases and 38 controls at T4, 20 cases and 28 controls at T5. Models are adjusted for age, BMI, physical activity, elevated blood pressure, and family history of type 2 diabetes. Dots represent mean concentrations and whiskers the 95% CI around the mean. HbA_1c_, Glycated haemoglobin; HDL, High‐density lipoprotein; GGT, Gamma‐glutamyltransferase; LDL, Low‐density lipoprotein; NA, not available; T, Time point; T_3_, Triiodothyronine; T_4,_ Thyroxine; T2DM, type 2 diabetes mellitus; TSH, Thyroid‐stimulating hormone. The Tromsø Study 1986–2016

Male cases experienced a significantly larger decrease in pre‐diagnostic total lipid, total cholesterol, and LDL concentrations compared to controls, whereas significantly larger increases in HbA_1c_ and glucose concentrations were observed from T2–T3 in cases (Figure 4 and Table S3). Further, there was a significantly larger increase in post‐diagnostic HbA_1c_ and HDL concentrations, and a larger decrease in free T_3_ concentrations in cases compared to controls from T3–T5.

### 3.3 Associations between pre‐diagnostic blood biomarker concentrations and T2DM

In women, pre‐diagnostic concentrations above the predefined cut‐offs for HDL (T1) and free T_4_ (T3) were inversely associated with T2DM, while total lipids and free T_3_ (T3); triglycerides, HbA_1c_ and glucose (T2 and T3); and GGT (T2) were positively associated with T2DM after adjusting for established risk factors (Table S4). Further, HDL (T3), HbA_1c_ (T2 and T3), GGT (T2), total lipids (T3), triglycerides (T3) and free T_3_ (T3) were associated with T2DM in a linear, dose‐response manner. For men, concentrations above the predefined cut‐offs for HbA_1c_ (T2 and T3), GGT (T2), total lipids, free T_3_ and non‐fasting glucose (T3) were positively associated with T2DM (Table S5). HbA_1c_ and glucose (T3) displayed a linear, dose‐response relationship with T2DM.

At T1, the established risk factors model showed a higher predictive ability than the blood biomarker model for both men and women, while at T2 and T3, the blood biomarker model performed better (Tables 2 and 3). However, the combined model had increased predictive ability at every pre‐diagnostic time point. The strongest discrimination between cases and controls was observed at T2 (95% for women and 85% for men), when the models for men and women were similar but not identical, as HDL was included for women only. Excluding HDL reduced discrimination among women to 94%, with a small loss of model fit (AIC 77.1 vs. 76.4).

**TABLE 2:** Multivariable prediction of type 2 diabetes according to established risk factors and blood biomarkers across pre‐diagnostic time points in women. The Tromsø Study 1986–2016

T1 (1986/87)			T2 (1994/95)			T3 (2001)		
	OR (95% CI)	AROC		OR (95% CI)	AROC		OR (95% CI)	AROC
Established risk factors^a^								
BMI ≥25 <30 (kg/m^2^)	3.19 (1.23, 8.27)	0.76	BMI ≥25 < 30 (kg/m^2^)	5.60 (1.70, 18.5)	0.83	BMI ≥25 < 30 (kg/m^2^)	4.46 (1.23, 16.2)	0.80
BMI ≥30 (kg/m^2^)	6.26 (1.86, 21.0)		BMI ≥30 (kg/m^2^)	15.3 (3.98, 58.7)		BMI ≥30 (kg/m^2^)	11.6 (3.05, 43.9)	
Physical activity (active)	1.20 (0.43, 3.34)		Physical activity (active)	0.36 (0.15, 0.90)		Physical activity (active)	0.40 (0.14, 1.12)	
Elevated blood pressure (yes)	2.19 (0.91, 5.27)		Elevated blood pressure (yes)	1.34 (0.48, 3.73)		Elevated blood pressure (yes)	2.91 (1.03, 8.19)	
Family history of T2DM (yes)	4.21 (1.28, 13.8)		Family history of T2DM (yes)	7.77 (2.40, 25.2)		Family history of T2DM (yes)	3.03 (1.12, 8.15)	
Blood biomarker^b^								
HDL ≥1.29 (mmol/L)	0.36 (0.16, 0.80)	0.66	Total lipids ≥7.40 (g/L)	3.88 (1.21, 12.5)	0.88	Triglycerides ≥1.70 (mmol/L)	5.69 (1.74, 18.7)	0.90
GGT ≥20.0 (U/L)	3.31 (1.12, 9.73)		HDL ≥1.29 (mmol/l)	0.47 (0.15, 1.47)		Free T_3_ ≥5.20 (pmol/L)	3.15 (0.77, 12.9)	
			HbA_1c_ ≥39.0 (mmol/moL)	15.2 (3.28, 70.8)		Free T_4_ ≥14.8 (pmol/L)	0.34 (0.10, 1.17)	
			GGT ≥20.0 (U/L)	18.3 (5.03, 66.4)		HbA_1c_ ≥39.0 (mmol/mol)	9.82 (3.03, 31.8)	
						Glucose ≥5.78 (mmol/L)	5.87 (1.68, 20.5)	
Combined model^a^, ^b^								
HDL ≥1.29 (mmol/L)	0.39 (0.16, 0.96)	0.79	HDL ≥1.29 (mmol/L)	0.29 (0.06, 1.35)	0.95	Triglycerides ≥1.70 (mmol/L)	7.28 (1.66, 31.9)	0.94
GGT ≥20.0 (U/L)	2.70 (0.81, 9.03)		HbA_1c_ ≥39.0 (mmol/mol)	39.7 (3.65, 432)		Free T_4_ ≥14.8 (pmol/L)	0.23 (0.05, 1.02)	
BMI ≥25 <30 (kg/m^2^)	3.06 (1.13, 8.25)		GGT ≥20.0 (U/l)	24.2 (3.65, 143)		HbA_1c_ ≥39.0 (mmol/mol)	13.3 (3.21, 55.0)	
BMI ≥30 (kg/m^2^)	4.52 (1.24, 16.5)		BMI ≥25 <30 (kg/m^2^)	11.4 (1.63, 79.8)		Glucose ≥5.78 (mmol/L)	3.25 (0.73, 14.4)	
Physical activity (active)	1.28 (0.43, 3.75)		BMI ≥30 (kg/m^2^)	16.9 (2.00, 142)		BMI ≥25 <30 (kg/m^2^)	1.00 (0.17, 5.94)	
Elevated blood pressure (yes)	2.12 (0.85, 5.27)		Physical activity (active)	0.19 (0.04, 0.85)		BMI ≥30 (kg/m^2^)	6.72 (1.08, 41.9)	
Family history of T2DM (yes)	3.91 (1.16, 13.2)		Elevated blood pressure (yes)	0.92 (0.15, 5.59)		Physical activity (active)	0.30 (0.06, 1.45)	
			Family history of T2DM (yes)	28.6 (3.66, 224)		Elevated blood pressure (yes)	3.22 (0.68, 15.3)	
						Family history of T2DM (yes)	2.25 (0.54, 9.36)	

Abbreviations: AROC, Area under the receiver operating characteristic curve; BMI, Body mass index; GGT, Gamma‐glutamyltransferase; HbA_1c_, Glycated haemoglobin; HDL, High‐density lipoprotein; T, Time point; T2DM, type 2 diabetes mellitus; T_3_, Triiodothyronine; T_4,_ Thyroxine.

^a^ Models were adjusted for age.

^b^ Models were selected with backwards selection process according to best model fit.

**TABLE 3:** Multivariable prediction of type 2 diabetes according to established risk factors and blood biomarkers across pre‐diagnostic time points in men. The Tromsø Study 1986–2016

T1 (1986/87)			T2 (1994/95)			T3 (2001)		
	OR (95%‐CI)	AROC		OR (95%‐CI)	AROC		OR (95%‐CI)	AROC
Established risk factors^a^								
BMI ≥25 <30 (kg/m^2^)	5.75 (2.31, 14.3)	0.73	BMI ≥25 < 30 (kg/m^2^)	3.59 (1.31, 9.84)	0.73	BMI ≥25 < 30 (kg/m^2^)	3.91 (1.16, 13.2)	0.71
BMI ≥0 (kg/m^2^)	8.02 (2.03, 31.7)		BMI ≥30 (kg/m^2^)	18.5 (4.34, 78.5)		BMI ≥30 (kg/m^2^)	13.6 (3.25, 56.5)	
Physical activity (active)	0.71 (0.23, 2.18)		Physical activity (active)	0.92 (0.36, 2.35)		Physical activity (active)	1.15 (0.43, 3.06)	
Elevated blood pressure (yes)	1.29 (0.50, 3.29)		Elevated blood pressure (yes)	0.96 (0.35, 2.62)		Elevated blood pressure (yes)	0.57 (0.19, 1.66)	
Family history of T2DM (yes)	1.47 (0.51, 4.25)		Family history of T2DM (yes)	1.09 (0.41, 2.88)		Family history of T2DM (yes)	1.10 (0.44, 2.77)	
Blood biomarker^b^								
Total lipids ≥7.59 (g/L)	2.51 (1.07, 5.89)	0.72	HbA_1c_ ≥39.0 (mmol/mol)	14.2 (2.81, 71.3)	0.78	Triglycerides ≥1.70 (mmol/l)	2.77 (1.04, 7.34)	0.79
Triglycerides ≥1.70 (mmol/l)	1.76 (0.75, 4.12)		GGT ≥25 (U/L)	14.4 (3.88, 53.1)		HbA_1c_ ≥39.0 (mmol/mol)	5.39 (2.11, 13.8)	
GGT ≥25 (U/L)	2.91 (1.25, 6.75)					Glucose ≥5.59 (mmol/L)	3.24 (1.22, 8.59)	
Combined model^a^, ^b^								
Total lipids ≥7.59 (g/L)	2.04 (0.81, 5.12)	0.78	HbA_1c_ ≥39.0 (mmol/mol)	12.9 (2.22, 75.3)	0.85	Triglycerides ≥1.70 (mmol/l)	3.93 (1.15, 13.4)	0.84
GGT ≥25 (U/l)	2.26 (0.89, 5.74)		GGT ≥25 (U/l)	11.6 (2.61, 51.6)		HbA_1c_ ≥39.0 (mmol/mol)	8.50 (2.59, 27.9)	
BMI ≥25 <30 (kg/m^2^)	3.89 (1.47, 10.3)		BMI ≥25 <30 (kg/m^2^)	2.08 (0.55, 7.77)		Glucose ≥5.59 (mmol/L)	3.17 (1.02, 9.81)	
BMI ≥30 (kg/m^2^)	3.96 (0.88, 17.7)		BMI ≥30 (kg/m^2^)	20.0 (1.61, 248)		BMI ≥25 <30 (kg/m^2^)	5.88 (1.25, 27.6)	
Physical activity (active)	0.72 (0.23, 2.28)		Physical activity (active)	2.23 (0.64, 7.81)		BMI ≥30 (kg/m^2^)	14.9 (2.47, 89.7)	
Elevated blood pressure (yes)	1.28 (0.49, 3.37)		Elevated blood pressure (yes)	0.55 (0.14, 2.19)		Physical activity (active)	1.26 (0.34, 4.64)	
Family history of T2DM (yes)	1.64 (0.56, 4.83)		Family history of T2DM (yes)	1.25 (0.34, 4.65)		Elevated blood pressure (yes)	0.49 (0.14, 1.73)	
						Family history of T2DM (yes)	1.44 (0.40, 5.25)	

Abbreviations: AROC, Area under the receiver operating characteristic curve; BMI, Body mass index; GGT, Gamma‐glutamyltransferase; HbA_1c_, Glycated haemoglobin; T, Time point; T2DM, type 2 diabetes mellitus.

^a^ Models were adjusted for age.

^b^ Models were selected with backwards selection process according to best model fit.

## 4 DISCUSSION

In this nested case–control study, we observed differences between cases and controls in total lipids, triglycerides, total cholesterol, HbA_1c_, glucose and GGT that were present 15 years before T2DM diagnosis in cases. The model including established risk factors (age, BMI, physical activity, blood pressure and family history of T2DM) was sufficient to acceptably discriminate between cases and controls as early as 15 years before diagnosis (AROC: 0.73 for men and 0.76 for women), but discrimination increased in the combined model, which added blood biomarkers (0.78 and 0.79, respectively). The blood biomarker model displayed better predictive ability than the established risk factor model 7 years before diagnosis in cases (T2, AROC: 0.78 versus 0.73 in men and 0.88 versus 0.83 in women), but the combined model gave excellent predictive ability for men (AROC: 0.85) and outstanding predictive ability for women (AROC: 0.95). These findings suggest that several biomarkers of metabolic homeostasis, alone or combined with basic clinical information, can be used to predict T2DM up to 7 years before diagnosis. These blood biomarkers can be analysed easily and cost‐effectively and provide objective measures. This approach could help identify high‐risk individuals early, allowing preventive interventions to be implemented.

Our results showed that, regardless of the pre‐diagnostic time point, a prediction model combining easily obtainable blood biomarkers and basic clinical information provided excellent predictive ability, even when different biomarkers are included. Using repeated measurements, we revealed that blood biomarkers have the potential to consistently predict disease 15 years before diagnosis. Our results are in agreement with other studies that used a single blood sample collected 5–10 years before T2DM diagnosis.^3^, ^20^, ^21^, ^22^, ^33^, ^34^ Although these studies included different basic clinical information, and sometimes different blood biomarkers, they all showed excellent discrimination (AROC: 0.78–0.90). They also displayed similar predictive abilities, although their biomarkers were different from ours, perhaps because their biomarkers were also related to prediabetic metabolic disturbances. For example, the prediction model proposed by the Framingham offspring study used personal information (age, sex, history of T2DM, BMI), blood pressure, HDL, triglycerides and fasting glucose and had excellent predictive ability (AROC: 0.85) 7 years before diagnosis.^20^ Our prediction model for women at T2 (also 7 years before diagnosis) was very similar (e.g., personal information, blood pressure, total lipids, triglycerides and HDL), but we included GGT and HbA_1c_, as fasting blood glucose was not available. As postprandial hyperglycaemia is more common in individuals with prediabetes,^35^, ^36^ fasting blood glucose may not identify disturbances in glucose homeostasis as well as HbA_1c_,^37^ which may also explain the higher predictive ability of our models compared to the Framingham model. Further, our results are based on non‐fasting blood samples, underlining the predictive value of non‐fasting biomarkers, which would alleviate some of the restrictions of risk models based on fasting blood samples. Our results also complement studies that included repeated measurements collected from patient's healthcare records in models for predicting T2DM. The studies by Paprott et al.^38^ and Pimentel et al.^39^ concluded that risk factors such as lifestyle habits, BMI/waist circumference, hypertension and family history of diabetes, as well as temporal changes in these risk factors, successfully predicted future T2DM. The studies by Gurka et al.^40^ and Bernardini et al.^41^ observed that concentrations and temporal changes in concentrations of triglycerides, HDL, LDL, GGT and urea, strengthened their prediction models.

In the present study, all prediction models performed better in women than in men. Specifically, we observed stronger associations between lipids (total lipids, triglycerides and HDL), free T_3_, free T_4_, HbA_1c_, glucose and T2DM in women than men. Several other studies (reviewed by Kautzky‐Willer et al.^42^) demonstrated stronger associations between lipids and incident T2DM in women than men, possibly due to sex differences in fat deposition.^42^ Njølstad et al.^43^ also observed stronger associations between HDL, triglycerides, random glucose and T2DM in women than men in the Finnmark Study; BMI was a more important risk factor for men.

Many blood biomarkers were significant predictors of T2DM in our study; however, discrimination and model fit were not compromised even after several biomarkers were excluded from the models. This may be due to the very strong predictive abilities of some blood biomarkers. For example, at T2, HDL was significantly associated with T2DM among women after adjusting for established risk factors, but discrimination and model fit did not improve significantly in a model that included only HbA_1c_, GGT and established risk factors. Unfortunately, we did not have GGT and HbA_1c_ at every pre‐diagnostic time point and could not include them together at T1 and T3. However, we hypothesize that, had they been available, their combined inclusion would have improved the model discrimination at these time points as well. This is in line with previous findings that HbA_1c_ and GGT were on par with or better than a combination of other blood lipids and/or glucose measurements and significantly improved discrimination beyond established risk factors.^3^, ^34^ As such, for clinical purposes, our study showed that the inclusion of HbA_1c,_ GGT and established risk factors would result in identical prediction models for men and women at all pre‐diagnostic time points with excellent predictive ability.

Already at T2, HbA_1c_ concentrations were significantly higher in cases (~37 mmol/mol, 5.5%) than controls (~35 mmol/mol, 5.4%), though they were still within normal limits (42–47 mmol/mol, 6.0–6.4%) according to the International Expert Committee.^44^ However, our results suggest that a lower HbA_1c_ threshold for risk assessment, one more in line with that recommended by the American Diabetes Association, may be warranted, as it would enable earlier identification of high‐risk subjects. Our results are in line with studies on HbA_1c_ trajectories, which showed similar differences between cases and controls up to 10 years before diagnosis (cases: 37.0–40.0 mmol/mol, 5.5–5.8%; controls: 33.0–35.5 mmol/mol, 5.2–5.4%).^45^, ^46^

We observed that cases had higher average GGT concentrations than controls and that men generally had higher concentrations than women. However, concentrations varied within the normal range of 10–75 U/l for women and 15–115 U/l for men.^31^ This is in line with previous studies investigating liver biomarkers in relation to T2DM, which showed significantly higher GGT concentrations in cases than controls, and in men than women, though they remained within normal limits.^5^, ^47^, ^48^ GGT has been identified as an independent risk factor for T2DM and is also linked to hepatic steatosis, which in turn is associated with obesity,^49^ clearly emphasizing the potential of GGT as a predictive biomarker for T2DM.

Total cholesterol and LDL concentrations decreased in both cases and controls throughout the study period. A general decrease in cholesterol concentrations in the Tromsø Study from 1979 to 2016 was previously reported for both men and women.^50^ The authors hypothesized that this was due to changes in cholesterol‐associated lifestyle factors in the Norwegian population, such as a general increase in physical activity, and decreased smoking and consumption of trans fats. In our study, the steeper post‐diagnostic decrease in cholesterol concentrations among cases may be explained by targeted lifestyle changes following the diagnosis, as individuals with T2DM have been shown to improve their lipid concentrations after diagnosis.^51^ The decrease may also be attributed to the use of cholesterol‐lowering drugs, as cardiovascular diseases are associated with T2DM. In our study, 43%–70% of cases and 5%–24% of controls reported using lipid‐lowering drugs at T4 and T5, compared to 17%–40% in the general population within similar age groups and time periods.^50^

We observed different changes in free T_3_ between cases and controls where cases generally had increased pre‐diagnostic and decreased post‐diagnostic concentrations. Free T_3_ was positively associated with T2DM in men and women at T3, whereas free T_4_ was inversely associated with T2DM in women at T3. This both agrees and disagrees with a recent meta‐analysis including 12 prospective studies^52^ that demonstrated positive associations between TSH concentrations and T2DM, and inverse associations between free T_3_ and free T_4_ with T2DM. We did not observe any significant associations between TSH and T2DM, possibly due to small sample size. Time of blood sampling before diagnosis as well as study design might explain the different study observations. Accordingly, we observed that concentrations of free T_3_ were similar between cases and controls at T1, with a notable increase in cases to T3, followed by a post‐diagnostic decline. This observation is in line with the study by Jun et al.^53^ where they observed an increased T_3_ concentration at baseline followed by a decline over time in cases. This highlights that repeated measurements are important especially due to the properties of thyroid hormone homeostasis regulated by feedback mechanisms.^54^, ^55^ Discrete alterations in thyroid hormones may not be detected by measurement from a single time point and the interrelationship between levels of TSH, free T_3_ and free T_4_ and their associations with T2DM can be dependent on timing of measurements. There are very few longitudinal studies with repeated measurements of thyroid hormones with which we can compare our results to, and to our knowledge, none have presented repeated free T_3_ measurements. Our observations may indicate an imbalance in thyroid homeostasis in T2DM cases, which may result in subclinical hyperthyroidism or hypothyroidism, and in turn, may affect insulin resistance and glucose concentrations.^56^

The main strength of this study is the nested case–control design with repeated measurements which allowed us to study pre‐ and post‐diagnostic changes over 30 years, and produce prediction models for the same individuals at three different pre‐diagnostic time points. Moreover, we had high‐quality information for many clinical variables, possible confounding factors and a wide spectrum of relevant biomarkers. The design provided us with an important evolutionary overview of the biomarkers and how they relate to the progression of T2DM and beyond. Information on T2DM diagnosis was collected from local registries and laboratory data up until the last survey, and medical records were used to confirm that none of the controls had been diagnosed with T2DM.

After stratifying by sex, there were few observations at each time point among cases and controls, which limits the precision of our effect estimates. Due to a lack of serum, we were not able to analyse thyroid hormones at T2 nor glucose at T1; moreover, GGT was unavailable at T3 and T5, as was HbA_1c_ at T1. Waist circumference was also not available at T1, and only available for ~68% of subjects at T2. However, even though waist circumference has a stronger association with T2DM than BMI, it has not been shown to provide more accurate risk predictions of T2DM.^57^ We had smaller sample sizes at post‐diagnostic time points, as the inclusion criteria required an available blood sample at all pre‐diagnostic ones. The prediction models were developed in a study sample from a northern Norwegian population, thus, the relative contribution of each predictor may vary in other populations due to genetical, environmental and lifestyle variations. Accordingly, our prediction models should be validated in different populations to verify their generalizability, and cut‐offs should be re‐evaluated if necessary.^19^

## 5 CONCLUSIONS

Already 15 years before diagnosis, there were clear differences in blood biomarker concentrations between T2DM cases and controls and several blood biomarkers were associated with type T2DM. Selected blood biomarkers (lipids, HbA_1c_, GGT) in combination with BMI, physical activity, elevated blood pressure, and family history of T2DM had excellent predictive ability 1–7 years before type 2 T2DM diagnosis and acceptable predictive ability up to 15 years before diagnosis.

## CONFLICT OF INTEREST

The authors have no conflict of interest to declare.

## AUTHOR CONTRIBUTIONS

**Giovanni Allaoui:** Data curation (lead); Formal analysis (lead); Methodology (lead); Writing – original draft (lead); Writing – review & editing (equal). **Charlotta Rylander:** Conceptualization (equal); Funding acquisition (supporting); Methodology (supporting); Project administration (equal); Supervision (supporting); Writing – original draft (supporting); Writing – review & editing (equal). **Maria Averina:** Supervision (supporting); Writing – original draft (supporting); Writing – review & editing (supporting). **Tom Wilsgaard:** Formal analysis (supporting); Methodology (supporting); Supervision (supporting); Writing – review & editing (supporting). **Ole‐Martin Fuskevåg:** Methodology (supporting); Supervision (supporting); Writing – review & editing (supporting). **Vivian Berg:** Conceptualization (equal); Funding acquisition (lead); Methodology (supporting); Project administration (lead); Resources (lead); Supervision (lead); Writing – original draft (supporting); Writing – review & editing (equal).

## Supporting information

Supplementary Material

## Data Availability

The data set used in present study was derived from the Tromsø Study. It is not publicly available, but may be accessed through an application to the Tromsø Study (https://uit.no/research/tromsostudy).
