# Supplementary material for: Longitudinal changes in blood biomarkers and their ability to predict type 2 diabetes mellitus—The Tromsø study
Source: Endocrinol Diabetes Metab. 2022 Feb 11;5(2):e00325. doi: 10.1002/edm2.325 (PMC8917864; doi:10.1002/edm2.325)
Supplement: Supplementary file 1 — Supplementary Material [file EDM2-5-e00325-s001.docx]

**Supplemental material**

**Longitudinal changes in blood biomarkers and their ability to predict Type 2 Diabetes Mellitus – The Tromsø study**

Giovanni Allaoui^ab^, Charlotta Rylander^c^, Maria Averina^ac^, Tom Wilsgaard^c^, Ole-Martin Fuskevåg^a^, Vivian Berg^ab*^

^a^Department of Laboratory Medicine, Division of Diagnostic Services, University Hospital of North-Norway, NO-9038 Tromsø, Norway

^b^Department of Medical Biology, Faculty of Health Sciences, UiT-The Arctic University of Norway, NO-9037 Tromsø, Norway

^c^Department of Community Medicine, Faculty of Health Sciences, UIT-The Arctic University of Norway, NO-9037 Tromsø, Norway

***Correspondence:**

Vivian Berg, Department of Medical Biology, Faculty of Health Sciences, UiT-The Arctic University of Norway, NO-9037 Tromsø, Norway

Phone: +47 77 64 46 77

Email: vivian.berg@uit.no

**Table S1**. Characteristics of the study sample across five surveys of the Tromsø Study 1986-2016.

|  |  |  | **Pre-diagnostic time-points** | | | **Post-diagnostic time-points** | |
| --- | --- | --- | --- | --- | --- | --- | --- |
|  |  |  | **T1**  **1986/87** | **T2**  **1994/95** | **T3**  **2001** | **T4**  **2007/08** | **T5**  **2015/16** |
| **Alcohol consumption, n (%)** |  |  |  |  |  |  |  |
| Teetotaller | Women  Men | Case  Control  Case  Control | 23 (50.0)  32 (47.8)  15 (29.4)  12 (20.3) | 14 (28.0)  27 (39.1)  10 (18.5)  4 (6.6) | 9 (18.4)  14 (20.6)  8 (15.4)  5 (8.20) | 10 (24.4)  10 (19.2)  6 (16.7)  4 (10.8) | 4 (15.4)  7 (18.0)  3 (15.0)  1 (3.6) |
| 1-4 times/ month | Women  Men | Case  Control  Case  Control | 21 (45.7)  34 (50.8)  31 (60.8)  39 (66.1) | 31 (62.0)  40 (58.0)  40 (74.1)  43 (70.5) | 33 (67.4)  41 (60.3)  34 (65.4)  41 (67.2) | 24 (58.5)  27 (51.9)  26 (72.2)  25 (67.6) | 17 (65.4)  22 (56.4)  14 (70.0)  16 (57.1) |
| > 4 times/ month | Women  Men | Case  Control  Case  Control | 2 (4.4)  1 (1.5)  5 (9.8)  8 (13.6) | 5 (10.0)  2 (2.9)  4 (7.4)  14 (23.0) | 7 (14.3)  13 (19.1)  10 (19.2)  15 (24.6) | 7 (17.1)  15 (28.9)  4 (11.1)  8 (21.6) | 5 (19.2)  10 (25.6)  3 (15.0)  11 (39.3) |
| **Physical activity, n (%)** |  |  |  |  |  |  |  |
| Inactive | Women  Men | Case  Control  Case  Control | 10 (20.0)  15 (21.7)  9 (16.7)  10 (16.4) | 30 (60.0)  22 (31.9)  16 (29.6)  15 (24.6) | 17 (34.7)  13 (19.4)  11 (21.2)  16 (27.1) | 10 (25.6)  5 (10.9)  9 (24.3)  8 (22.2) | 6 (26.1)  3 (8.8)  10 (52.6)  2 (7.7) |
| Active | Women  Men | Case  Control  Case  Control | 40 (80.0)  54 (78.3)  45 (83.3)  51 (83.6) | 20 (40.0)  47 (68.1)  38 (70.4)  46 (75.4) | 32 (65.3)  54 (80.6)  41 (78.9)  43 (72.9) | 29 (74.4)  41 (89.1)  28 (75.7)  28 (77.7) | 17 (73.9)  31 (91.2)  9 (47.4)  24 (92.3) |
| **Family history of type 2 diabetes, n (%)** |  |  |  |  |  |  |  |
| No | Women  Men | Case  Control  Case  Control | 37 (74.0)  63 (91.3)  43 (79.6)  51 (83.6) | 30 (60.0)  61 (88.4)  41 (75.9)  48 (78.7) | 29 (58.0)  56 (81.2)  38 (70.4)  46 (75.4) | 28 (63.6)  42 (79.3)  26 (68.4)  29 (76.3) | 16 (61.5)  34 (85.0)  13 (65.0)  23 (82.1) |
| Yes | Women  Men | Case  Control  Case  Control | 13 (26.0)  6 (8.70)  11 (20.4)  10 (16.4) | 20 (40.0)  8 (11.6)  13 (24.1)  13 (21.3) | 21 (42.0)  13 (18.8)  16 (29.6)  15 (24.6) | 16 (36.4)  11 (20.8)  12 (31.6)  9 (23.7) | 10 (38.5)  6 (15.0)  7 (35.0)  5 (17.9) |
| **Elevated blood pressure, n (%)** |  |  |  |  |  |  |  |
| No | Women  Men | Case  Control  Case  Control | 19 (38.0)  46 (66.7)  12 (22.2)  20 (32.8) | 16 (32.0)  32 (46.4)  10 (18.5)  16 (26.2) | 10 (20.0)  31 (44.9)  11 (20.4)  14 (23.0) | 3 (6.82)  15 (28.3)  5 (13.2)  10 (26.3) | 3 (11.5)  10 (25.0)  3 (15.0)  7 (25.0) |
| Yes | Women  Men | Case  Control  Case  Control | 31 (62.0)  23 (33.3)  42 (77.8)  41 (67.2) | 34 (68.0)  37 (53.6)  44 (81.5)  45 (73.8) | 40 (80.0)  38 (55.1)  43 (79.6)  47 (77.1) | 41 (93.2)  38 (71.7)  33 (86.8)  28 (73.7) | 23 (88.5)  30 (75.0)  17 (85.0)  21 (75.0) |
| **Us of lipid-lowering drugs, n (%)** |  |  |  |  |  |  |  |
| No | Women  Men | Case  Control  Case  Control | NA | 49 (98.0)  68 (98.6)  51 (94.4)  61 (100) | 41 (85.4)  60 (88.3)  38 (73.1)  54 (91.5) | 25 (56.8)  43 (82.7)  20 (52.6)  36 (94.7) | 10 (40.0)  26 (76.5)  6 (30.0)  20 (76.9) |
| Yes | Women  Men | Case  Control  Case  Control | NA | 1 (1.45)  1 (2.00)  3 (5.56)  0 (0.00) | 7 (14.6)  8 (11.8)  14 (26.9)  5 (8.47) | 19 (43.2)  9 (17.3)  18 (47.4)  2 (5.26) | 15 (60.0)  8 (23.5)  14 (70.0)  6 (23.1) |

T: time-point.

Table S2. Pre- and post- diagnostic blood biomarker concentrations across five surveys in The Tromsø Study 1986-2016.

|  |  |  | **Pre-diagnostic time-points** | | | | | | **Post-diagnostic time-points** | | | |
| --- | --- | --- | --- | --- | --- | --- | --- | --- | --- | --- | --- | --- |
|  |  |  | **T1**  **1986/87** | | **T2**  **1994/95** | | **T3**  **2001** | | **T4**  **2007/08** | | **T5**  **2015/16** | |
| **Biomarker** |  |  | **Mean (SD)** | **Median (5, 95 percentiles)** | **Mean (SD)** | **Median (5, 95 percentiles)** | **Mean (SD)** | **Median (5, 95 percentiles)** | **Mean (SD)** | **Median (5, 95 percentiles)** | **Mean (SD)** | **Median (5, 95 percentiles)** |
| Total lipids  (g/L) | Women^a^  Men^b^ | Case  Control  Case  Control | 7.52 (1.49)  6.91 (1.42)  8.47 (2.03)  7.42 (1.34) | 7.48 (5.43, 9.98)  6.88 (4.81, 9.40)  8.23 (5.86, 12.2)  7.45 (5.22, 9.65) | 8.31 (2.10)  7.22 (1.42)  8.20 (1.64)  8.04 (2.61) | 7.97 (5.87, 10.7)  7.21 (4.88, 9.77)  8.00 (5.95, 11.4)  7.48 (5.56, 10.8) | 7.73 (1.28)  6.98 (1.20)  7.37 (1.30)  7.19 (1.43) | 7.59 (5.82, 9.79)  6.98 (5.04, 9.01)  7.56 (5.03, 9.53)  7.16 (5.03, 8.90) | 7.38 (1.50)  7.09 (1.27)  7.09 (1.04)  7.43 (1.45) | 7.40 (5.01, 9.87)  6.86 (5.40, 9.16)  6.99 (5.67, 8.83)  6.81 (5.80, 10.3) | 6.51 (1.32)  6.61 (0.86)  6.16 (1.78)  6.54 (1.60) | 6.13 (5.30, 9.22)  6.71 (5.46, 7.75)  6.06 (4.03, 10.7)  6.04 (4.75, 9.31) |
| Triglycerides  (mmol/L) | Women  Men | Case  Control  Case  Control | 1.69 (1.02)  1.19 (0.55)  2.33 (1.64)  1.71 (0.84) | 1.36 (0.69, 4.28)  1.00 (0.59, 2.30)  1.97 (0.94, 5.44)  1.51 (0.73, 3.22) | 1.83 (2.10)  1.31 (0.62)  2.27 (1.24)  2.10 (1.93) | 1.67 (0.76, 3.62)  1.12 (0.68, 2.67)  1.95 (1.05, 4.99)  1.70 (0.81, 4.12) | 1.98 (0.94)  1.35 (0.58)  2.03 (0.94)  1.65 (1.10) | 1.78 (0.77, 3.66)  1.18 (0.72, 2.74)  1.92 (0.97, 4.07)  1.54 (0.74, 2.82) | 2.12 (0.85)  1.34 (0.52)  2.20 (0.88)  1.86 (1.08) | 1.81 (1.30, 4.19)  1.21 (0.65, 2.31)  2.02 (1.14, 4.01)  1.75 (0.61, 4.05) | 2.01 (1.02)  1.34 (0.51)  1.81 (0.88)  1.54 (0.92) | 1.63 (0.97, 3.50)  1.21 (0.70, 2.34)  1.78 (0.82, 3.96)  1.32 (0.68, 3.84) |
| Total cholesterol  (mmol/L) | Women  Men | Case  Control  Case  Control | 6.16 (1.02)  5.96 (1.26)  6.59 (1.28)  6.02 (1.07) | 6.09 (4.70, 7.94)  5.91 (3.76, 7.92)  6.69 (4.60, 9.00)  5.98 (4.25, 8.02) | 6.69 (1.21)  6.20 (1.22)  6.33 (1.04)  6.33 (1.32) | 6.84 (5.03, 8.84)  6.18 (4.09, 8.09)  6.29 (4.54, 8.32)  6.20 (4.36, 8.12) | 6.11 (0.95)  5.89 (1.05)  5.63 (0.95)  5.82 (1.07) | 6.31 (4.39, 7.48)  5.94 (4.20, 7.49)  5.60 (3.99, 7.00)  5.67 (4.24, 7.95) | 5.56 (1.21)  6.01 (1.07)  5.15 (0.80)  5.88 (1.24) | 5.61 (3.67, 7.08)  5.84 (4.61, 7.87)  5.03 (4.18, 6.57)  5.56 (4.27, 8.04) | 4.68 (0.81)  5.47 (0.95)  4.48 (1.30)  5.19 (1.13) | 4.56 (3.48, 5.94)  5.47 (4.07, 6.71)  4.43 (2.76, 7.45)  4.95 (3.85, 6.84) |
| LDL  (mmol/L) | Women  Men | Case  Control  Case  Control | 4.06 (0.96)  4.01(1.10)  4.43 (1.34)  4.12 (1.01) | 4.03 (2.40, 5.58)  4.03 (2.17, 5.69)  4.50 (2.38, 6.91)  4.06 (2.53, 5.76) | 4.32 (1.09)  4.01 (1.06)  4.00 (1.04)  4.02 (0.97) | 4.22 (2.80, 6.36)  3.88 (2.18, 5.64)  3.90 (2.43, 6.05)  4.06 (2.44, 5.53) | 3.97 (0.90)  3.78 (0.99)  3.64 (0.92)  3.92 (1.00) | 4.00 (2.59, 5.41)  3.78 (2.22, 5.54)  3.72 (2.11, 4.96)  3.79 (2.56, 5.64) | 3.40 (0.93)  3.86 (1.07)  3.23 (0.73)  4.02 (1.21) | 3.43 (1.67, 5.01)  3.71 (2.39, 5.88)  3.20 (2.41, 4.68)  3.81 (2.54, 6.24) | 2.61 (0.69)  3.30 (0.89)  2.65 (1.04)  3.34 (0.93) | 2.61 (1.56, 3.70)  3.26 (2.09, 4.78)  2.64 (1.42, 4.94)  3.33 (1.95, 4.78) |
| HDL  (mmol/L) | Women  Men | Case  Control  Case  Control | 1.37 (0.41)  1.52 (0.33)  1.08 (0.26)  1.15 (0.36) | 1.30 (0.86, 2.07)  1.45 (1.09, 2.13)  1.08 (0.69, 1.56)  1.07 (0.72, 1.65) | 1.39 (0.39)  1.54 (0.33)  1.07 (0.23)  1.11 (0.37) | 1.31 (0.9, 2.24)  1.50 (1.03, 2.15)  1.04 (0.67, 1.38)  1.08 (0.57, 1.71) | 1.32 (0.37)  1.60 (0.41)  1.13 (0.27)  1.27 (0.40) | 1.32 (0.84, 2.04)  1.58 (0.92, 2.32)  1.10 (0.7, 1.71)  1.19 (0.76, 1.95) | 1.46 (0.52)  1.76 (0.42)  1.13 (0.29)  1.20 (0.29) | 1.38 (0.90, 2.24)  1.73 (1.12, 2.60)  1.13 (0.79, 1.65)  1.18 (0.80, 1.74) | 1.39 (0.41)  1.78 (0.50)  1.23 (0.37)  1.31 (0.25) | 1.36 (0.76, 2.14)  1.72 (0.93, 2.58)  1.08 (0.82, 2.12)  1.30 (0.85, 1.70) |
| Free T_3_  (pmol/L) | Women  Men | Case  Control  Case  Control | 4.88 (0.55)  4.81 (0.54)  5.33 (0.56)  5.23 (0.60) | 4.82 (3.84, 5.60)  4.81 (4.06, 5.53)  5.29 (4.38, 6.29)  5.24 (4.37, 6.32) | NA | NA | 5.15 (1.30)  4.64 (0.54)  5.10 (0.53)  4.85 (0.85) | 4.93 (4.06, 6.73)  4.65 (3.78, 5.50)  5.05 (4.30, 6.12)  4.93 (4.03, 6.03) | 4.82 (0.57)  4.71 (0.53)  5.22 (0.53)  5.11 (0.50) | 4.89 (4.01, 5.60)  4.60 (3.83, 5.78)  5.22 (4.52, 6.20)  5.00 (4.46, 6.00) | 4.44 (0.63)  4.58 (0.45)  4.51 (0.35)  4.90 (0.63) | 4.34 (3.71, 5.78)  4.50 (3.90, 5.27)  4.45 (3.98, 5.16)  4.83 (4.15, 6.15) |
| Free T_4_  (pmol/L) | Women  Men | Case  Control  Case  Control | 14.1 (2.00)  14.5 (2.39)  14.6 (1.95)  15.0 (2.31) | 13.6 (11.2, 18.0)  14.4 (10.9, 19.0)  14.5 (11.5, 18.4)  15.0 (11.1, 18.1) | NA | NA | 14.3 (3.30)  14.5 (2.30)  14.4 (2.37)  14.9 (2.44) | 14.2 (11.1, 17.2)  14.6 (11.6, 17.9)  13.8 (10.8, 19.2)  14.8 (11.5, 19.3) | 14.6 (1.87)  15.4 (2.08)  15.3 (1.43)  14.7 (2.02) | 14.5 (11.8, 18.0)  15.3 (11.8, 18.8)  15.2 (12.9, 17.5)  14.7 (11.7, 17.0) | 15.7 (2.01)  15.7 (2.27)  16.0 (2.22)  16.1 (2.97) | 15.5 (13.1, 18.2)  15.2 (13.5, 20.8)  15.4 (12.7, 20.1)  15.6 (12.3, 22.8) |
| TSH  (mIU/L) | Women  Men | Case  Control  Case  Control | 1.92 (0.95)  2.22 (2.34)  2.17 (1.01)  2.18 (1.14) | 1.87 (0.82, 3.83)  1.60 (0.66, 4.12)  2.03 (0.64, 4.08)  1.89 (0.70, 4.11) | 1.71 (0.88)  2.15 (2.50)  1.43 (0.71)  1.81 (1.14) | 1.41 (0.71, 3.41)  1.51 (0.89, 3.20)  1.33 (0.66, 3.14)  1.50 (0.48, 3.45) | 1.94 (0.97)  2.30 (2.73)  2.17 (1.00)  2.37 (1.34) | 1.85 (0.75, 3.61)  1.81 (0.75, 4.96)  1.96 (0.74, 3.81)  2.10 (0.81, 5.06) | 2.04 (0.97)  1.93 (0.86)  2.14 (1.19)  2.20 (0.82) | 1.89 (0.89, 3.68)  1.70 (0.77, 3.71)  2.03 (0.55, 4.80)  2.23 (0.92, 3.61) | 1.92 (0.74)  2.19 (1.95)  1.91 (0.86)  2.43 (0.83) | 1.83 (0.98, 3.47)  1.74 (0.52, 6.48)  1.82 (0.31, 3.55)  2.48 (1.16, 3.74) |
| Hba1c  (mmol/mol) | Women  Men | Case  Control  Case  Control | NA | NA | 37.1 (4.99)  34.9 (3.41)  37.2 (3.57)  35.2 (3.22) | 37.7 (30.0, 43.2)  35.0 (30.0, 41.0)  36.6 (33.3, 42.1)  35.5 (30.0, 39.9) | 40.5 (4.76)  34.5 (4.71)  40.1 (4.66)  35.6 (4.39) | 40.1 (32.1, 47.2)  33.4 (27.2, 43.4)  40.0 (32.2, 46.9)  35.8 (27.3, 43.2) | 50.7 (12.8)  38.9 (3.81)  48.8 (7.52)  37.4 (3.42) | 47.0 (39.9, 65.0)  39.9 (33.3, 45.3)  48.6 (37.7, 61.7)  37.7 (31.1, 42.1) | 52.4 (8.72)  39.5 (3.03)  51.3 (6.65)  38.0 (2.94) | 49.7 (42.1, 69.4)  39.9 (34.4, 45.3)  50.8 (41.5, 65.6)  37.7 (32.2, 42.1) |
| Glucose  (mmol/L) | Women  Men | Case  Control  Case  Control | NA | NA | 5.28 (0.73)  4.68 (0.57)  4.91 (0.57)  4.78 (0.71) | 5.25 (4.40, 6.50)  4.70 (3.80, 5.60)  4.80 (4.20, 5.90)  4.90 (3.70, 6.10) | 6.46 (2.35)  5.28 (0.78)  6.53 (3.17)  5.37 (0.76) | 5.86 (4.39, 10.3)  5.17 (4.25, 6.58)  5.89 (4.47, 9.74)  5.39 (4.17, 6.48) | 6.53 (3.42)  5.02 (0.58)  6.85 (1.52)  5.17 (0.49) | 5.70 (4.70, 10.9)  4.85 (4.40, 6.10)  6.60 (5.1, 10.2)  5.20 (4.30, 6.00) | 7.54 (2.98)  5.31 (0.56)  7.19 (2.19)  5.59 (0.99) | 6.70 (4.60, 13.5)  5.20 (4.30, 6.80)  6.75 (4.30, 12.3)  5.40 (4.70, 7.20) |
| GGT  (U/L) | Women  Men | Case  Control  Case  Control | 22.1 (23.4)  13.6 (6.03)  30.7 (26.1)  23.7 (24.0) | 13.5 (7.00, 90.0)  13.0 (7.00, 28.0)  24.5 (10.0, 70.0)  17.5 (8.00, 49.0) | 40.8 (41.9)  20.3 (18.3)  40.5 (28.2)  29.2 (28.7) | 23.5 (12.0, 109)  16.0 (9.00, 47.0)  32.0 (14.0, 84.0)  21.0 (12.0, 57.0) | NA | NA | 44.0 (47.7)  26.4 (21.4)  53.0 (39.8)  27.2 (20.3) | 26.0 (13.0, 138)  19.5 (8.00, 84.0)  35.0 (13.0, 139)  21.0 (12.5, 82.5) | NA | NA |

^a^50 cases and 69 controls at T1-T3, 44 cases and 53 controls at T4, 26 cases and 40 controls at T5.

^b^54 cases and 61 controls at T1-T3, 38 cases and 38 controls at T4, 20 cases and 28 controls at T5.

T: time-point; TSH: thyroid stimulating hormone; GGT: gamma-glutamyltransferase.

**Table S3.** Multivariable adjusted regression coefficients, standard error, and 95% CI from linear mixed effect models to assess longitudinal changes in biomarkers from 1986 to 2016 according to type 2 diabetes mellitus status. Tromsø Study 1986-2016.

| **Biomarker** |  | **Males^a^** | | | **Females^b^** | | |
| --- | --- | --- | --- | --- | --- | --- | --- |
|  |  | **β-coefficient**  **(SE)** | **p-value** | **95% confidence interval** | **β-coefficient**  **(SE)** | **p-value** | **95% confidence interval** |
| **Total lipids**  **(g/L)** | Case  T1  T2  T3  T4  T5  Case#T1  Case#T2  Case#T3  Case#T4  Case#T5  Constant | -0.32 (0.27)  0.62 (0.29)  1.00 (0.26)  Reference  0.35 (0.22)  -0.70 (0.32)  0.91 (0.32)  0.02 (0.36)  Reference  -0.72 (0.32)  -0.67 (0.41)  2.76 (1.14) | 0.24  0.03  <0.01  --  0.12  0.03  <0.01  0.96  --  0.02  0.10  0.02 | -0.85, 0.21  0.06, 1.19  0.49, 1.51  --  -0.09, 0.78  -1.33, -0.08  0.29, 1.53  -0.68, 0.72  --  -1.34, -0.10  -1.48, 0.14  0.52, 4.99 | 0.52 (0.23)  0.87 (0.22)  0.68 (0.21)  Reference  -0.22 (0.28)  -1.06 (0.29)  -0.12 (0.26)  0.33 (0.30)  Reference  -0.37 (0.41)  -0.84 (0.41)  3.03 (0.70) | 0.03  <0.01  <0.01  --  0.43  <0.01  0.65  0.27  --  0.37  0.04  <0.01 | 0.07, 0.98  0.43, 1.31  0.27, 1.08  --  -0.76, 0.32  -1.64, -0.49  -0.63, 0.39  -0.26, 0.91  --  -1.16, 0.43  -1.64, -0.04  1.65, 4.41 |
| **Triglycerides**  **(mmol/L)** | Case  T1  T2  T3  T4  T5  Case#T1  Case#T2  Case#T3  Case#T4  Case#T5  Constant | 0.06 (0.20)  0.16 (0.22)  0.47 (0.20)  Reference  0.30 (0.19)  0.12 (0.20)  0.27 (0.24)  -0.17 0.28)  Reference  -0.21 (0.27)  -0.46 (0.23)  -0.62 (0.84) | 0.76  0.47  0.02  --  0.12  0.56  0.27  0.53  --  0.44  0.05  0.46 | -0.34, 0.46  -0.27, 0.59  0.08, 0.86  --  -0.07, 0.67  -0.27, 0.50  -0.21, 0.74  -0.71, 0.37  --  -0.74, 0.32  -0.91, -0.01  -2.27, 1.02 | 0.42 (0.15)  0.11 (0.14)  0.08 (0.10)  Reference  -0.05 (0.13)  -0.21 (0.17)  -0.08 (0.16)  -0.10 (0.13)  Reference  0.12 (0.19)  0.08 (0.22)  -0.24 (0.44) | 0.01  0.41  0.41  --  0.69  0.21  0.63  0.44  --  0.54  0.71  0.59 | 0.12, 0.72  -0.16, 0.39  -0.11, 0.27  --  -0.31, 0.21  -0.54, 0.12  -0.38, 0.23  -0.36, 0.16  --  -0.26, 0.49  -0.35, 0.52  -1.11, 0.63 |
| **Total Cholesterol**  **(mmol/L)** | Case  T1  T2  T3  T4  T5  Case#T1  Case#T2  Case#T3  Case#T4  Case#T5  Constant | -0.44 (0.20)  0.56 (0.20)  0.67 (0.15)  Reference  0.09 (0.17)  -0.83 (0.25)  0.77 (0.21)  0.19 (0.19)  Reference  -0.56 (0.24)  -0.40 (0.33)  3.02 (0.81) | 0.02  0.01  <0.01  --  0.57  <0.01  <0.01  0.32  --  0.02  0.23  <0.01 | -0.83, -0.06  0.17, 0.95  0.38, 0.95  --  -0.23, 0.42  -1.32, -0.35  0.35, 1.18  -0.18, 0.57  --  -1.02, -0.1  -1.07, 0.26  1.42, 4.61 | 0.09 (0.19)  0.95 (0.18)  0.72 (0.15)  Reference  -0.20 (0.23)  -1.03 (0.25)  -0.03 (0.21)  0.27 (0.21)  Reference  -0.58 (0.33)  -1.10 (0.36)  2.49 (0.55) | 0.61  <0.01  <0.01  --  0.37  <0.01  0.87  0.20  --  0.08  <0.01  <0.01 | -0.27, 0.46  0.60, 1.30  0.43, 1.01  --  -0.64, 0.24  -1.52, -0.53  -0.45, 0.38  -0.14, 0.68  --  -1.23, 0.07  -1.79, -0.40  1.41, 3.56 |
| **LDL**  **(mmol/L)** | Case  T1  T2  T3  T4  T5  Case#T1  Case#T2  Case#T3  Case#T4  Case#T5  Constant | -0.47 (0.19)  0.35 (0.19)  0.17 (0.12)  Reference  0.08 (0.16)  -0.66 (0.20)  0.61 (0.21)  0.28 (0.16)  Reference  -0.47 (0.22)  -0.22 (0.27)  2.15 (0.75) | 0.01  0.07  0.18  --  0.62  <0.01  <0.01  0.08  --  0.03  0.43  <0.01 | -0.84, -0.11  -0.02, 0.73  -0.07, 0.41  --  -0.23, 0.38  -1.06, -0.26  0.21, 1.01  -0.03, 0.58  --  -0.90, -0.04  -0.75, 0.32  0.67, 3.63 | 0.09 (0.18)  0.86 (0.17)  0.53 (0.14)  Reference  -0.17 (0.20)  -0.93 (0.22)  -0.15 (0.20)  0.11 (0.19)  Reference  -0.55 (0.29)  -0.94 (0.31)  1.35 (0.52) | 0.60  <0.01  <0.01  --  0.40  <0.01  0.45  0.58  --  0.06  <0.01  0.01 | -0.26, 0.44  0.53, 1.19  0.26, 0.80  --  -0.56, 0.23  -1.37, -0.49  -0.53, 0.24  -0.28, 0.49  --  -1.12, 0.03  -1.56, -0.32  0.33, 2.37 |
| **HDL**  **(mmol/L)** | Case  T1  T2  T3  T4  T5  Case#T1  Case#T2  Case#T3  Case#T4  Case#T5  Constant | -0.06 (0.06)  -0.09 (0.05)  -0.14 (0.03)  Reference  -0.02 (0.04)  -0.06 (0.05)  0.05 (0.04)  0.08 (0.04)  Reference  0.04 (0.05)  0.13 (0.06)  1.61 (0.22) | 0.37  0.06  <0.01  --  0.70  0.29  0.19  0.05  --  0.47  0.05  <0.01 | -0.18, 0.05  -0.19, 0.02  -0.20, -0.06  --  -0.09, 0.05  -0.16, 0.07  -0.03, 0.14  0.001, 0.16  --  -0.07, 0.19  0.001, 0.25  1.17, 2.04 | -0.20 (0.08)  -0.06 (0.07)  -0.06 (0.04)  Reference  0.12 (0.06)  0.11 (0.08)  0.09 (0.06)  0.12 (0.05)  Reference  0.04 (0.08)  -0.12 (0.09)  1.83 (0.25) | 0.01  0.38  0.18  --  0.04  0.14  0.12  0.02  --  0.64  0.19  <0.01 | -0.35, -0.04  -0.20, 0.07  -0.14, 0.03  --  0.003, 0.23  -0.04, 0.27  -0.02, 0.21  0.02, 0.21  --  -0.12, 0.20  -0.29, 0.06  1.34, 2.32 |
| **Free T3**  **(pmol/L)** | Case  T1  T3  T4  T5  Case#T1  Case#T3  Case#T4  Case#T5  Constant | 0.19 (0.13)  0.12 (0.11)  Reference  0.25 (0.12)  0.16 (0.10)  -0.16 (0.13)  Reference  -0.20 (0.17)  -0.60 (0.13)  5.24 (0.42) | 0.16  0.30  --  0.04  0.12  0.24  --  0.24  <0.01  <0.01 | -0.07, 0.45  -0.11, 0.34  --  0.01, 0.48  -0.04, 0.36  -0.42, 0.11  --  -0.54, 0.14  -0.85, -0.35  4.43, 6.06 | 0.47 (0.18)  -0.06 (0.14)  Reference  0.13 (0.14)  0.09 (0.14)  -0.43 (0.19)  Reference  -0.46 (0.21)  -0.51 (0.18)  5.49 (0.36) | 0.01  0.66  --  0.37  0.53  0.02  --  0.03  0.01  <0.01 | 0.11, 0.83  -0.34, 0.22  --  -0.15, 0.40  -0.18, 0.35  -0.80, -0.06  --  -0.87, -0.05  -0.87, -0.16  4.78, 6.20 |
| **Free T4**  **(pmol/L)** | Case  T1  T3  T4  T5  Case#T1  Case#T3  Case#T4  Case#T5  Constant | 0.31 (0.44)  -1.15 (0.35)  Reference  0.40 (0.28)  2.37 (0.47)  -0.13 (0.29)  Reference  0.73 (0.39)  0.02 (0.59)  23.2 (1.61) | 0.49  <0.01  --  0.15  <0.01  0.66  --  0.06  0.97  <0.01 | -0.56, 1.18  -1.83, -0.47  --  -0.14, 0.94  1.44, 3.29  -0.70, 0.44  --  -0.04, 1.49  -1.14, 1.19  20.1, 26.4 | -0.18 (0.53)  -0.35 (0.44)  Reference  0.72 (0.39)  1.08 (0.46)  -0.14 (0.49)  Reference  -0.35 (0.56)  0.61 (0.57)  15.8 (1.46) | 0.73  0.43  --  0.07  0.02  0.78  --  0.53  0.29  <0.01 | -1.21, 0.85  -1.22, 0.52  --  -0.05, 1.48  0.18, 1.96  -1.10, 0.82  --  -1.44, 0.74  -0.52, 1.73  13.0, 18.7 |
| **TSH**  **(mIU/L)** | Case  T1  T2  T3  T4  T5  Case#T1  Case#T2  Case#T3  Case#T4  Case#T5  Constant | -0.20 (0.23)  0.02 (0.18)  -0.51 (0.16)  Reference  -0.11 (0.17)  0.04 (0.20)  0.13 (0.18)  -0.08 (0.22)  Reference  0.04 (0.23)  -0.44 (0.27)  1.40 (0.77) | 0.39  0.90  <0.01  --  0.51  0.84  0.46  0.71  --  0.87  0.10  0.07 | -0.65, 0.25  -0.33, 0.38  -0.83, -0.18  --  -0.44, 0.22  -0.35, 0.43  -0.22, 0.49  -0.52, 0.35  --  -0.50, 0.42  -0.97, 0.09  -0.12, 2.91 | -0.29 (0.27)  0.51 (0.26)  -0.23 (0.18)  Reference  -0.18 (0.18)  -0.20 (0.32)  -0.10 (0.23)  0.07 (0.24)  Reference  0.23 (0.23)  -0.47 (0.38)  0.24 (0.94) | 0.29  0.05  0.20  --  0.31  0.55  0.66  0.78  --  0.33  0.22  0.80 | -0.82, 0.25  0.002, 1.02  -0.58, 0.12  --  -0.52, 0.17  -0.83, 0.44  -0.54, 0.34  -0.41, 0.55  --  -0.23, 0.68  -1.21, 0.27  -1.61, 2.08 |
| **HbA1c (mmol/mol)** | Case  T2  T3  T4  T5  Case#T2  Case#T3  Case#T4  Case#T5  Constant | 4.42 (0.99)  -0.39 (0.78)  Reference  1.82 (1.05)  2.14 (1.12)  -2.52 (1.08)  Reference  7.28 (1.49)  8.32 (1.60)  33.7 (3.44) | <0.01  0.62  --  0.08  0.06  -4.64  --  4.36  5.18  <0.01 | 2.49, 6.35  -1.91, 1.14  --  -0.24, 3.89  -0.05, 4.33  -4.64, -0.41  --  4.36, 10.2  5.18, 11.5  27.0, 40.5 | 5.75 (1.04)  1.22 (0.73)  Reference  3.50 (1.52)  3.84 (1.26)  -3.98 (1.01)  Reference  6.03 (2.24)  7.29 (1.83)  24.1 (3.27) | <0.01  0.10  --  0.02  <0.01  <0.01  --  0.01  <0.01  <0.01 | 3.72, 7.79  -0.22, 2.66  --  0.51, 6.48  1.36, 6.32  -5.96, -2.01  --  1.63, 10.4  3.71, 10.9  17.7, 30.5 |
| **Glucose (mmol/L)** | Case  T2  T3  T4  T5  Case#T2  Case#T3  Case#T4  Case#T5  Constant | 0.98 (0.43)  -0.57 (0.28)  Reference  -0.18 (0.31)  0.24 (0.45)  -0.94 (0.41)  Reference  0.52 (0.45)  0.55 (0.67)  4.61 (0.80) | 0.02  0.04  --  0.57  0.59  0.02  --  0.24  0.42  <0.01 | 0.15, 1.82  -1.12, -0.02  --  -0.78, 0.43  -0.64, 1.12  -1.73, -0.14  --  -0.35, 1.39  -0.77, 1.86  3.06, 6.17 | 0.96 (0.31)  -0.66 (0.21)  Reference  -0.18 (0.41)  0.18 (0.40)  -0.49 (0.31)  Reference  0.53 (0.61)  1.43 (0.61)  5.68 (0.62) | <0.01  <0.01  --  0.67  0.65  0.11  --  0.39  0.02  <0.01 | 0.36, 1.57  -1.07, -0.25  --  -0.99, 0.63  -0.60, 0.97  -1.10, 0.12  --  -0.67, 1.73  0.24, 2.61  4.47, 6.89 |
| **GGT (U/L)** | Case  T1  T2  T4  Case#T1  Case#T2  Case#T4  Constant | 1.91 (5.40)  -1.43 (3.34)  Reference  -3.14 (5.76)  -3.72 (3.94)  Reference  10.7 (7.06)  -46.0 (20.7) | 0.72  0.67  --  0.59  0.35  --  0.13  0.03 | -8.68, 12.5  -7.97, 5.11  --  -14.4, 8.16  -11.5, 4.01  --  -3.18, 24.5  -86.5, -5.48 | 16.7 (5.99)  -4.82 (3.06)  Reference  6.30 (7.11)  -10.8 (4.20)  Reference  -3.53 (9.80)  -2.05 (12.0) | 0.01  0.12  --  0.38  0.01  --  0.72  0.86 | 4.95, 28.4  -10.8, 1.17  --  -7.63, 20.2  -19.1, -2.61  --  -22.7, 15.7  -25.6, 21.4 |

^a^50 cases and 69 controls at T1-T3, 44 cases and 53 controls at T4, 26 cases and 40 controls at T5.

^b^54 cases and 61 controls at T1-T3, 38 cases and 38 controls at T4, 20 cases and 28 controls at T5.

Models adjusted for BMI, age, elevated blood pressure, physical activity, and type 2 diabetes family history.

T: time-point; TSH: thyroid stimulating hormone; GGT: gamma-glutamyltransferase.

Table S4. OR and 95% CI for the associations between pre-diagnostic blood biomarker concentrations and incident type 2 diabetes in females. The Tromsø Study 1986-2016.

| **Females** | **T1**  **1986/87** | | **T2**  **1994/95** | | **T3**  **2001** | |
| --- | --- | --- | --- | --- | --- | --- |
|  | **Crude** | **Adjusted^a^** | **Crude** | **Adjusted^a^** | **Crude** | **Adjusted^a^** |
| **Biomarker** | **OR**  **(95% CI)** | **OR**  **(95% CI)** | **OR**  **(95% CI)** | **OR**  **(95% CI)** | **OR**  **(95% CI)** | **OR**  **(95% CI)** |
| **Total lipids ≥7.40 g/L** | 2.07  (0.98, 4.34) | 1.49  (0.58, 3.82) | 3.42  (1.58, 7.40)* | 1.86  (0.66, 5.23) | 3.56  (1.66, 7.63)* | 4.68  (1.66, 13.2)* |
| **Total lipids (g/L)** | 1.34  (1.03, 1.73)* | 1.21  (0.85, 1.73) | 1.51  (1.16, 1.97)* | 1.49  (1.08, 2.07)* | 1.63  (1.19, 2.25)* | 1.69  (1.12, 2.54)* |
| **Triglycerides ≥1.70 mmol/L** | 2.42  (1.05, 5.56)* | 1.61  (0.61, 4.29) | 3.63  (1.62, 8.13)* | 2.82  (1.02, 7.78)* | 5.43  (2.41, 12.2)* | 4.81  (1.80, 12.8)* |
| **Triglycerides (mmol/L)** | 2.39  (1.35, 4.24)* | 1.77  (0.92, 3.42) | 2.56  (1.48, 4.45)* | 1.95  (1.00, 3.78)* | 3.24  (1.76, 5.95)* | 3.02  (1.53, 5.98)* |
| **Total cholesterol ≥5.00 mmol/L** | 1.58  (0.62, 4.06) | 1.07  (0.27, 4.28) | 7.35  (0.90, 60.0) | 3.99  (0.41, 39.1) | 2.01  (0.76, 5.29) | 1.87  (0.60, 5.85) |
| **Total Cholesterol (mmol/L)** | 1.16  (0.84, 1.59) | 1.03  (0.64, 1.66) | 1.40  (1.02, 1.92)* | 1.38  (0.89, 2.16) | 1.24  (0.86, 1.80) | 1.21  (0.76, 1.95) |
| **HDL ≥1.29 mmol/L** | 0.33  (0.15, 0.72)* | 0.37  (0.16, 0.95)* | 0.35  (0.16, 0.76)* | 0.51  (0.20, 1.32) | 0.30  (0.13, 0.67)* | 0.47  (0.18, 1.25) |
| **HDL (mmol/L)** | 0.30  (0.10, 0.87)* | 0.49  (0.14, 1.64) | 0.28  (0.10, 0.81)* | 0.61  (0.17, 2.21) | 0.15  (0.05, 0.45)* | 0.21  (0.06, 0.74)* |
| **LDL ≥3.00 mmol/L** | 1.22  (0.46, 3.20) | 0.71  (0.18, 2.83) | 1.35  (0.42, 4.30) | 0.51  (0.10, 2.56) | 1.85  (0.73, 4.68) | 1.02  (0.32, 3.27) |
| **LDL (mmol/L)** | 1.05  (0.74, 1.49) | 0.91  (0.55, 1.52) | 1.31  (0.93, 1.86) | 1.25  (0.76, 2.06) | 1.24  (0.84, 1.83) | 1.15  (0.70, 1.86) |
| **Free T_3_ ≥5.20** | 1.19  (0.52, 2.71) | 0.99  (0.38, 2.57) | NA | NA | 3.62  (1.50, 8.72)* | 3.30  (1.14, 9.57)* |
| **Free T3 (pmol/L)** | 1.28  (0.65, 2.52) | 1.01  (0.45, 2.28) | NA | NA | 2.72  (1.36, 5.46)* | 2.39  (1.01, 5.66)* |
| **Free T_4_ ≥14.8** | 0.53  (0.24, 1.13) | 0.47  (0.19, 1.14) | NA | NA | 0.31  (0.14, 0.70)* | 0.26  (0.10, 0.72)* |
| **Free T4 (pmol/L)** | 0.92  (0.78, 1.09) | 0.92  (0.77, 1.11) | NA | NA | 0.97  (0.85, 1.11) | 0.95  (0.81, 1.12) |
| **TSH ≥1.92** | 1.53  (0.73, 3.19) | 1.14  (0.49, 2.66) | 1.41  (0.36, 5.49) | 0.19  (0.01, 2.63) | 1.25  (0.60, 2.60) | 1.03  (0.43, 2.49) |
| **TSH (mIU/L)** | 0.90  (0.70, 1.16) | 0.80  (0.52, 1.22) | 0.87  (0.54, 1.39) | 0.68  (0.24, 1.95) | 0.90  (0.71, 1.15) | 0.82  (0.51, 1.32) |
| **HbA1c ≥39.0 mmol/mol** | NA | NA | 5.08  (1.65, 15.6)* | 6.16  (1.42, 26.7)* | 14.7  (4.93, 44.0)* | 15.7  (4.66, 53.1)* |
| **HbA1c (mmol/mol)** | NA | NA | 1.14  (1.02, 1.26)* | 1.15  (1.00, 1.32)* | 1.29  (1.16, 1.43)* | 1.31  (1.14, 1.51)* |
| **Glucose ≥5.78 mmol/L** | NA | NA | 6.81  (1.39, 33.3)* | 55.4  (1.26, 187)* | 8.48  (3.46, 20.8)* | 4.23  (1.49, 12.0)* |
| **Glucose (mmol/L)** | NA | NA | 5.01  (2.21, 11.4)* | 5.37  (1.86, 15.5)* | 2.34  (1.47, 3.73)* | 1.74  (1.06, 2.84)* |
| **GGT ≥20.0 U/L** | 3.63  (1.27, 10.4)* | 2.77  (0.85, 9.10) | 6.43  (2.86, 14.4)* | 5.10  (1.95, 13.4)* | NA | NA |
| **GGT (U/L)** | 1.05  (1.01, 1.09)* | 1.04  (0.99, 1.08) | 1.03  (1.01, 1.05)* | 1.02  (1.00, 1.04)* | NA | NA |

^a^Adjusted for age, BMI, physical activity, elevated blood pressure, and family history of type 2 diabetes. TSH: thyroid stimulating hormone; GGT: gamma-glutamyltransferase; *p<0.05

Table S5. OR and 95% CI for the associations between pre-diagnostic blood biomarker concentrations and incident type 2 diabetes in males. The Tromsø Study 1986-2016.

| **Males** | **T1**  **1986/87** | | **T2**  **1994/95** | | **T3**  **2001** | |
| --- | --- | --- | --- | --- | --- | --- |
|  | **Crude** | **Adjusted^a^** | **Crude** | **Adjusted^a^** | **Crude** | **Adjusted^a^** |
| **Biomarker** | **OR**  **(95% CI)** | **OR**  **(95% CI)** | **OR**  **(95% CI)** | **OR**  **(95% CI)** | **OR**  **(95% CI)** | **OR**  **(95% CI)** |
| **Total lipids ≥7.59 g/L** | 3.20  (1.47, 6.93)* | 1.99  (0.82, 4.82) | 2.05  (0.96, 4.36) | 1.26  (0.53, 3.00) | 2.81  (1.29, 6.14)* | 3.31  (1.37, 7.99)* |
| **Total lipids (g/L)** | 1.53  (1.16, 2.03)* | 1.26  (0.93, 1.72) | 1.03  (0.87, 1.22) | 0.93  (0.75, 1.16) | 1.10  (0.84, 1.44) | 0.92  (0.67, 1.27) |
| **Triglycerides ≥1.70 mmol/L** | 2.88  (1.34, 6.17)* | 2.11  (0.88, 5.07) | 1.57  (0.73, 3.34) | 0.84  (0.34, 2.11) | 2.29  (1.08, 4.84)* | 1.91  (0.77, 4.75) |
| **Triglycerides (mmol/L)** | 1.69  (1.11, 2.57)* | 1.26  (0.79, 2.01) | 1.06  (0.84, 1.34) | 0.96  (0.73, 1.26) | 1.49  (0.98, 2.26) | 1.17  (0.77, 1.77) |
| **Total cholesterol ≥5.00 mmol/L** | 1.48  (0.53, 4.13) | 0.98  (0.29, 3.25) | 1.69  (0.53, 5.42) | 0.78  (0.19, 3.21) | 0.70  (0.29, 1.68) | 0.43  (0.15, 1.21) |
| **Total Cholesterol (mmol/L)** | 1.52  (1.09, 2.12)* | 1.28  (0.88, 1.86) | 1.00  (0.73, 1.37) | 0.83  (0.56, 1.23) | 0.83  (0.58, 1.20) | 0.70  (0.44, 1.11) |
| **HDL ≥1.03 mmol/L** | 1.23  (0.59, 2.59) | 1.90  (0.78, 4.66) | 0.82  (0.38, 1.74) | 1.58  (0.63, 3.96) | 0.56  (0.25, 1.23) | 0.83  (0.33, 2.12) |
| **HDL (mmol/L)** | 0.49  (0.14, 1.66) | 0.82  (0.19, 3.48) | 0.65  (0.19, 2.15) | 1.33  (0.32, 5.51) | 0.28  (0.09, 0.91)* | 0.61  (0.16, 2.34) |
| **LDL ≥3.00 mmol/L** | 0.97  (0.38, 2.50) | 0.82  (0.28, 2.40) | 1.08  (0.42, 2.74) | 0.73  (0.25, 2.14) | 0.46  (0.19, 1.14) | 0.36  (0.11, 1.18) |
| **LDL (mmol/L)** | 1.25  (0.91, 1.73) | 1.17  (0.81, 1.69) | 0.98  (0.68, 1.43) | 0.86  (0.54, 1.35) | 0.73  (0.50, 1.09) | 0.62  (0.38, 1.02) |
| **Free T_3_ ≥5.12** | 1.44  (0.68, 3.05) | 1.40  (0.58, 3.37) | NA | NA | 2.23  (1.03, 4.84)* | 3.09  (1.18, 8.09)* |
| **Free T3 (pmol/L)** | 1.35  (0.72, 2.56) | 1.33  (0.60, 2.98) | NA | NA | 1.81  (0.95, 3.45) | 2.27  (0.90, 5.76) |
| **Free T_4_ ≥14.0** | 0.54  (0.25, 1.16) | 0.84  (0.33, 2.12) | NA | NA | 0.56  (0.27, 1.17) | 0.77  (0.34, 1.79) |
| **Free T4 (pmol/L)** | 0.92  (0.78, 1.10) | 1.08  (0.86, 1.35) | NA | NA | 0.92  (0.79, 1.07) | 1.06  (0.87, 1.29) |
| **TSH ≥1.85** | 1.44  (0.68, 3.05) | 1.10  (0.48, 2.53) | 0.42  (0.11, 1.60) | 0.53  (0.10, 2.87) | 1.08  (0.51, 2.27) | 1.10  (0.47, 2.56) |
| **TSH (mIU/L)** | 0.99  (0.70, 1.39) | 0.79  (0.52, 1.20) | 0.63  (0.32, 1.22) | 0.57  (0.22, 1.43) | 0.87  (0.63, 1.19) | 0.91  (0.62, 1.34) |
| **HbA1c ≥39.0 mmol/mol** | NA | NA | 4.81  (1.43, 16.3)* | 4.59  (1.15, 18.3)* | 6.61  (2.70, 16.2)* | 8.58  (2.96, 24.9)* |
| **HbA1c (mmol/mol)** | NA | NA | 1.20  (1.04, 1.37)* | 1.15  (0.98, 1.34)* | 1.25^a^  (1.12, 1.39)* | 1.27  (1.11, 1.45)* |
| **Glucose ≥5.59 mmol/L** | NA | NA | 1.39  (0.43, 4.52) | 1.39  (0.35, 5.64) | 3.47  (1.61, 7.50)* | 3.29  (1.38, 7.81)* |
| **Glucose (mmol/L)** | NA | NA | 1.35  (0.71, 2.57) | 1.15  (0.54, 2.46) | 1.98  (1.28, 3.05)* | 1.96  (1.19, 3.21)* |
| **GGT ≥25.0 U/L** | 3.36  (1.51, 7.47)* | 2.28  (0.91, 5.71) | 4.86  (2.17, 10.9)* | 3.13  (1.24, 7.94)* | NA | NA |
| **GGT (U/L)** | 1.01  (1.00, 1.03)* | 1.00  (0.98, 1.02) | 1.02  (1.00, 1.04)* | 1.00  (0.99, 1.02) | NA | NA |

^a^Adjusted for age, BMI, physical activity, elevated blood pressure, and family history of type 2 diabetes. TSH: thyroid stimulating hormone; GGT: gamma-glutamyltransferase; *p<0.05
